# Supplementary material for: Discovery of Anti-Coronavirus Cinnamoyl Triterpenoids Isolated from Hippophae rhamnoides during a Screening of Halophytes from the North Sea and Channel Coasts in Northern France
Source: Int J Mol Sci. 2023 Nov 22;24(23):16617. doi: 10.3390/ijms242316617 (PMC10705938; doi:10.3390/ijms242316617)

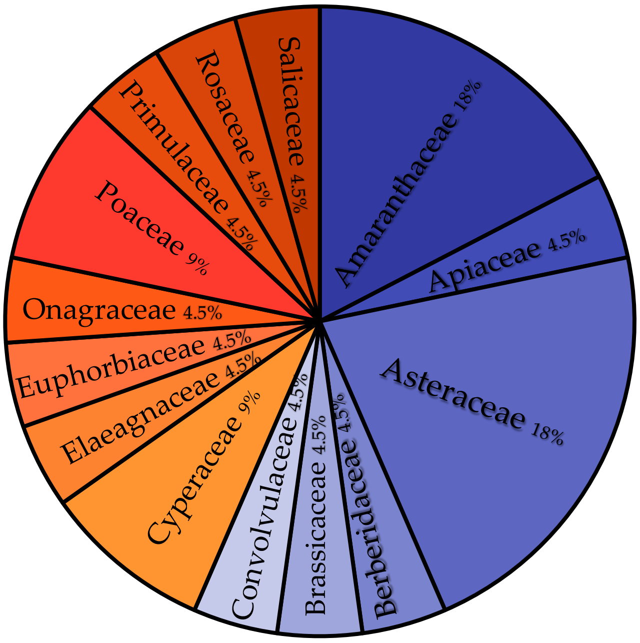

Figure S1. Chart representing the percentage of the collected plants botanical families.

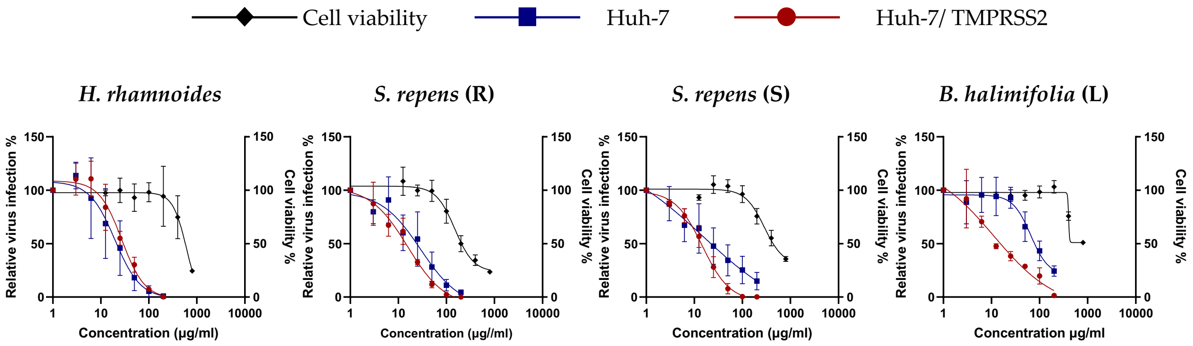

Figure S2. Cytotoxicity and antiviral activity on HCoV-229E of crude methanolic extracts of *Hippophae rhamnoides*, *Salix repens* (R), *Salix repens* (S), and *Baccharis halimifolia* (L). For infection assays, Huh-7 cells were inoculated with HCoV-229E in presence of various concentrations of each crude methanolic extract up to 200 µg/ml for 7 h. Cells were lysed 7 h post-inoculation and luciferase activity quantified. For cytotoxicity assays, cells were incubated with the different crude methanolic extracts at different concentrations, up to 800 µg/ml for 24 h. MTS assay was performed to monitor cell viability. Results are expressed as mean ± SEM of 3 independent experiments.

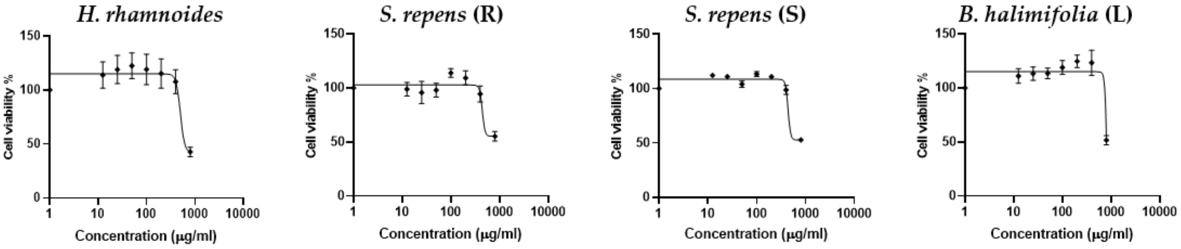

Figure S3. Cytotoxicity of crude methanolic extracts of *Hippophae rhamnoides*, Salix repens (R), Salix repens (S), and *Baccharis halimifolia* (L). Vero-81 cells were incubated with the different crude methanolic extracts at different concentrations, up to 800 µg/ml for 24 h. MTS assay was performed to monitor cell viability. Results are expressed as mean ± SEM of 3 independent experiments.

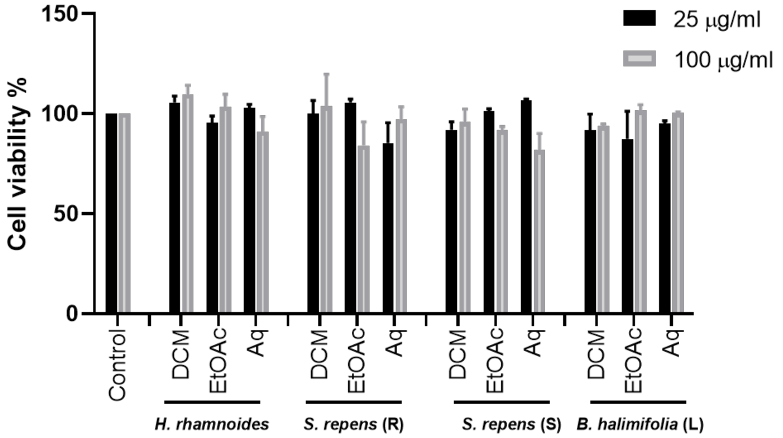

Figure S4. Effect of the different sub-extracts on Huh-7 cell viability. Cells were treated with the different sub-extracts for 24 h at 25 and 100 µg/ml or with 0.1% DMSO (control). No significant difference between the sub-extracts and control ($P < 0.05$). Data are represented as mean ± SEM of three independent experiments.

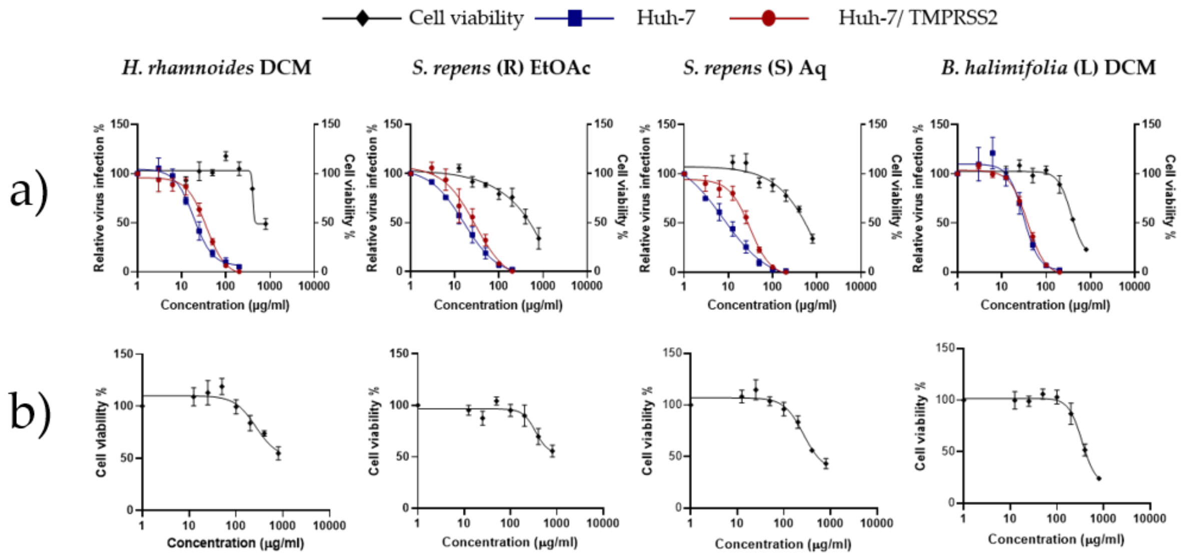

Figure S5. Cytotoxicity and antiviral activity on HCoV-229E of sub extracts of *Hippophae rhamnoides* DCM, *Salix repens* (R) EtOAc, *Salix repens* (S) Aq, and *Baccharis halimifolia* (L) DCM **a)** Cell viability and inhibition of HCoV-229E infection of Huh-7 cells in the presence of increasing concentrations of the sub-extracts. The infection was quantified by measuring luciferase activity **b)** Dose-response curves showing cell viability as a function of sub-extracts concentration, measured with the MTS assay in Vero-81 cells, after 24h. Data points are mean ± SEM.

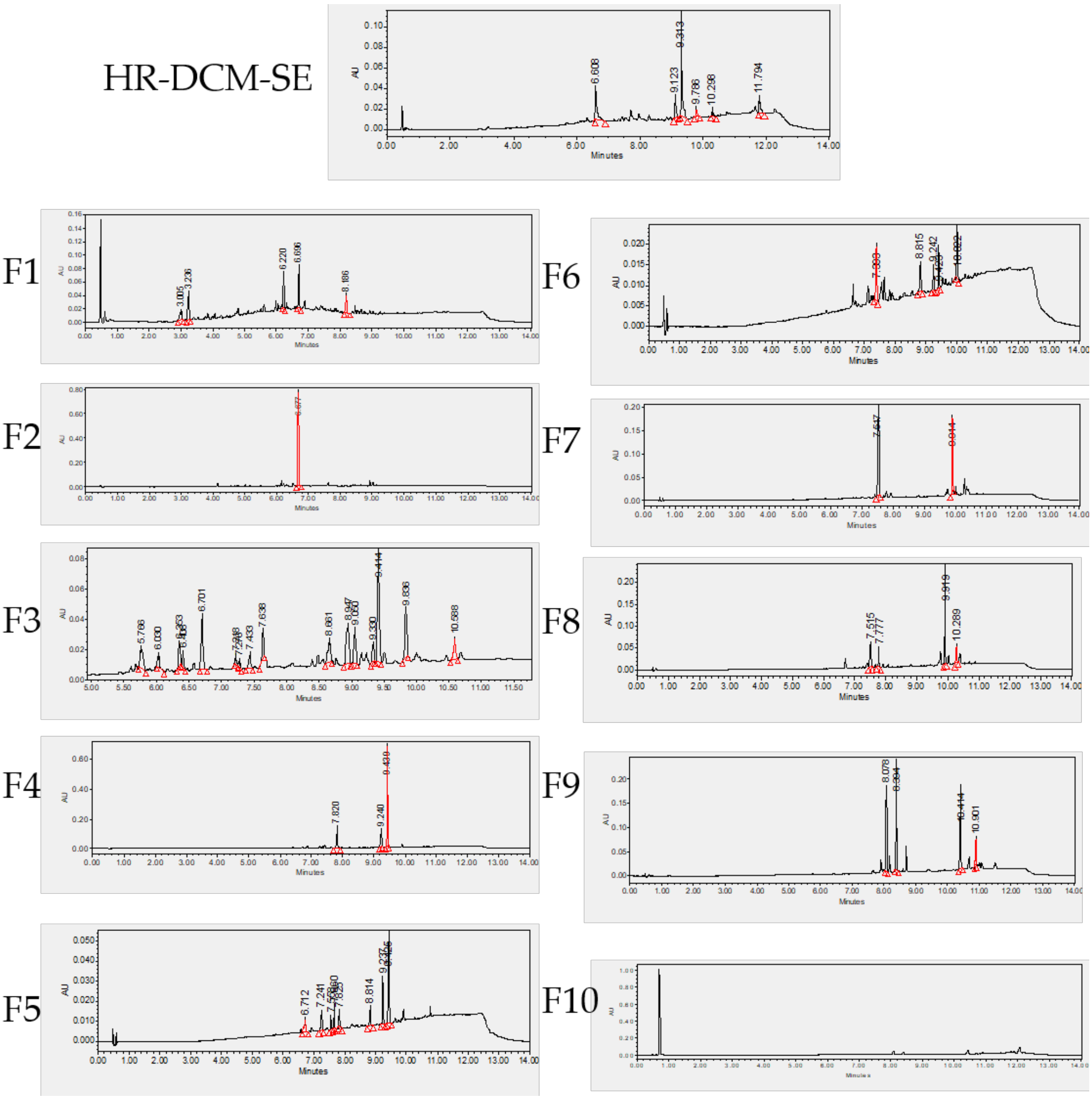

Figure S6. Chromatograms obtained by UHPLC-UV-MS at λ = 254 nm of different fractions resulting from CPC fractionation of the DCM sub-extract of *Hippophae rhamnoides*.

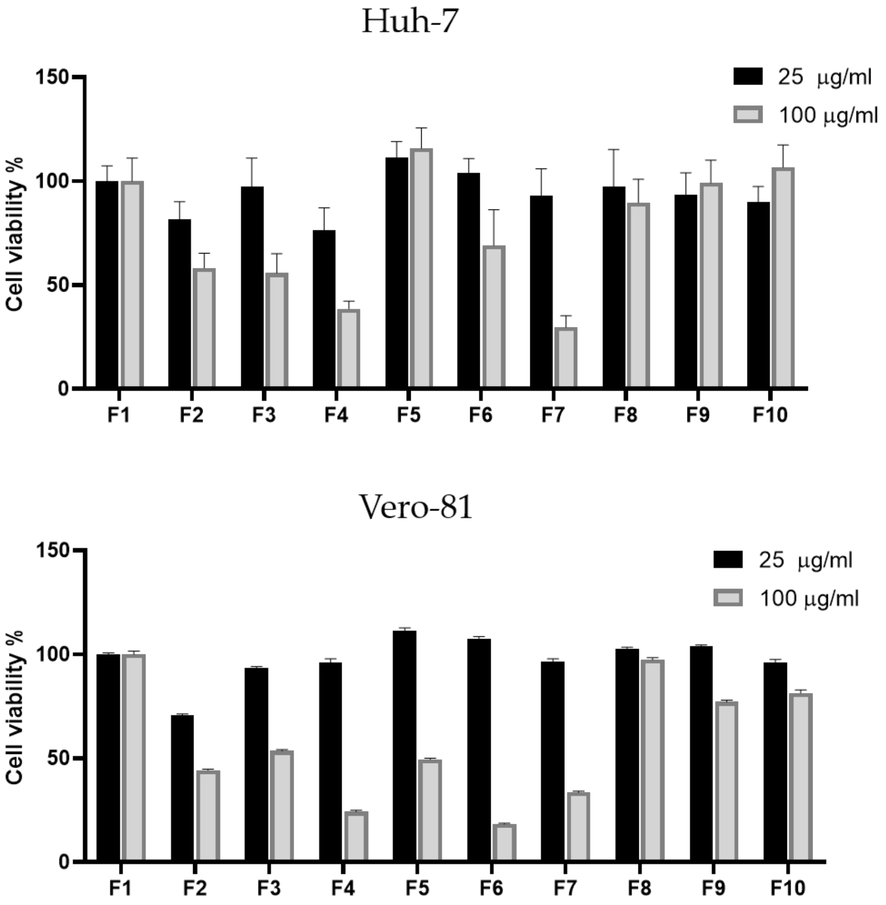

Figure S7. Cytotoxicity of fractions resulting from CPC fractionation of the DCM sub-extract of *Hippophae rhamnoides*. Effect of the different fractions on Huh-7 and Vero-81 cell viability when treated for 24 h at 25 and 100 µg/ml. Data are represented as mean ± SEM of three independent experiments.

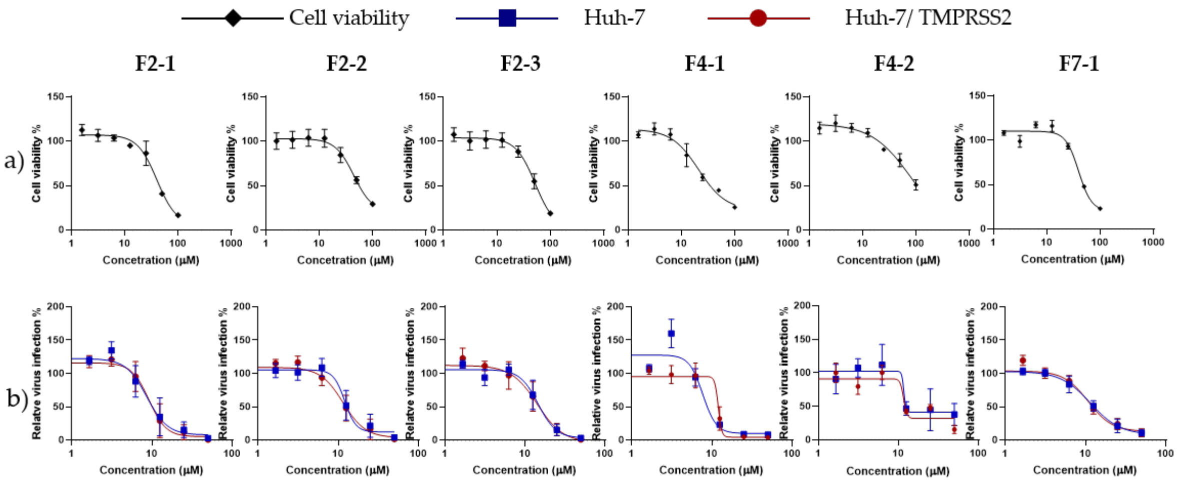

Figure S8. Cytotoxicity and antiviral activity on HCoV-229E of different pure compounds. **a)** Dose-response curves showing cell viability as a function of pure compounds concentrations, measured with the MTS assay in Huh-7 cells, after 24 h. Data points are mean ± SEM. **b)** Inhibition of HCoV-229E infection of Huh-7 cells in the presence of increasing concentrations of different pure compounds. The infection was quantified by measuring luciferase activity.

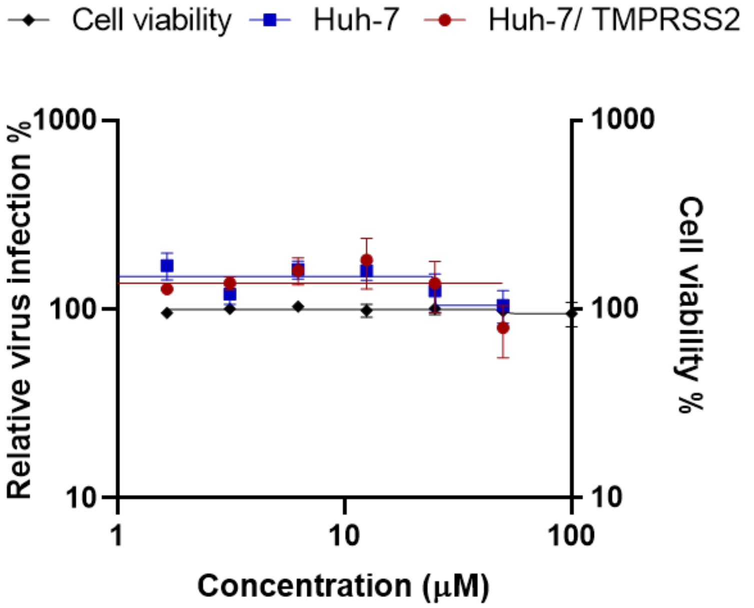

Figure S9. Cytotoxicity and HCoV-229E infectivity assays of F2-4 isolated from F2 of *Hippophae rhamnoides* DCM SE. For infection assays, Huh-7 cells were inoculated with HCoV-229E in presence of various concentrations of each crude methanolic extract up to 50 µM for 7 h. Cells were lysed 7 h post-inoculation and luciferase activity quantified. For toxicity assays, cells were incubated with the different concentrations, up to 800 µM for 24 h. MTS assay was performed to monitor cell viability. Results are expressed as mean ± SEM of 3 independent experiments.

Figure S10. Purity of cinnamoyl triterpenoids isolated from DCM sub-extract of *Hippohae rhamnoides* on the basis of PDA chromatograms

(F2-1) 2-*O*-trans-*p*-coumaroylmaslinic acid

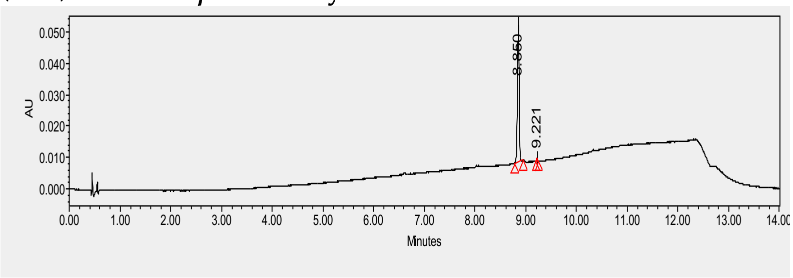

	Name	Retention Time	Area	% Area	Height
1		8.850	99425	**98.81**	43699
2		9.221	1199	1.19	788

(F2-2) 3β-hydroxy-2α-*trans*-*p*-coumaryloxy-urs-12-en-28-oic acid

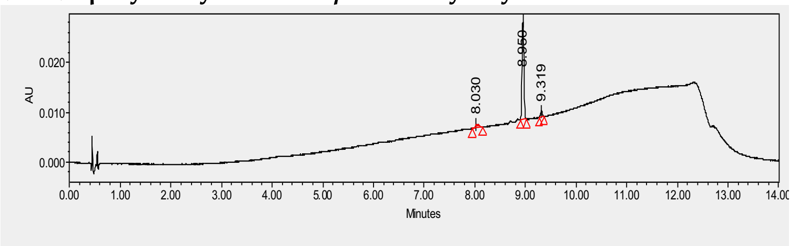

	Name	Retention Time	Area	% Area	Height
1		8.030	3263	5.69	739
2		8.950	50829	**88.63**	19645
3		9.319	3255	5.68	1318

(F2-3) 3β-hydroxy-2α-cis-*p*-coumaryloxy-urs-12-en-28-oic acid

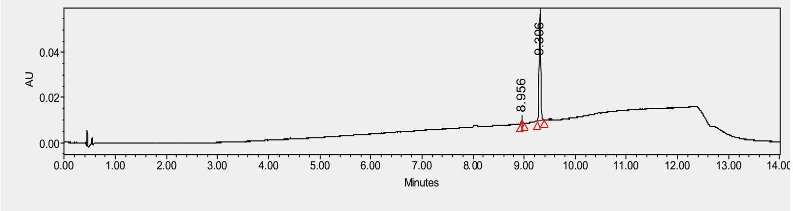

	Name	Retention Time	Area	% Area	Height
1		8.956	3109	2.86	1299
2		9.306	105645	**97.14**	47118

(F4-1) Mixture 3-*O*-*trans*-caffeoyl oleanolic acid / 3-*O*-*cis*-caffeoyl oleanolic acid (70/30)

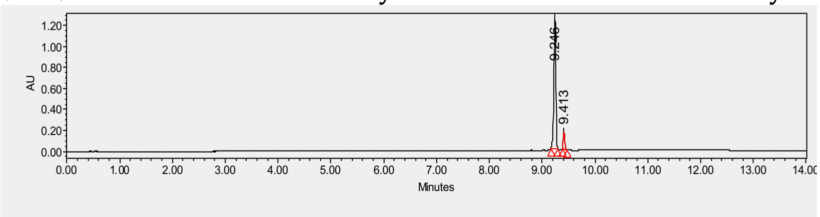

	Name	Retention Time	Area	% Area	Height
1		9.246	3197867	**90.71**	1228338
2		9.413	327318	9.29	159315

(F4-2) Oleanolic acid caffeate = 3-*O*-*trans*-caffeoyl oleanolic acid

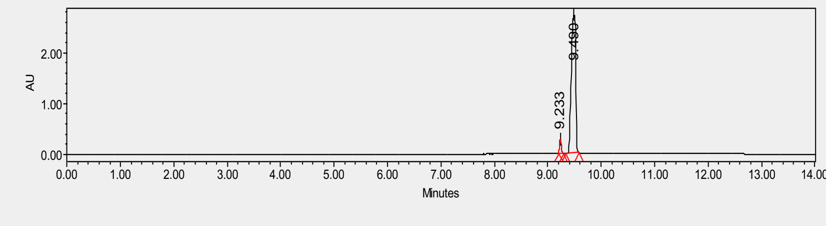

	Name	Retention Time	Area	% Area	Height
1		9.233	591875	3.64	284740
2		9.490	15686611	**96.36**	2716586

(F7-1) 3-O-*trans*-*p*-coumaroyl oleanolic acid

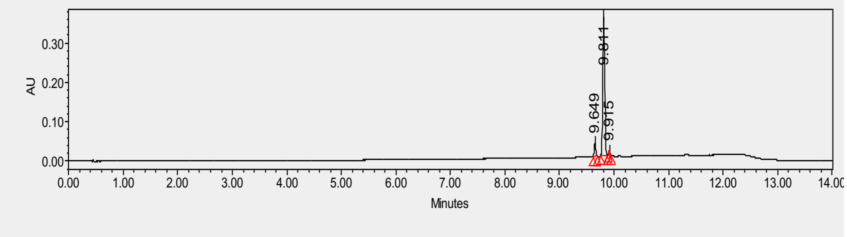

	Name	Retention Time	Area	% Area	Height
1		9.649	69990	7.26	34654
2		9.811	884184	**91.68**	353043
3		9.915	10293	1.07	7280

Figure S11. NMR data of cinnamoyl triterpenoids isolated from DCM sub-extract of *Hippophae rhamnoides*

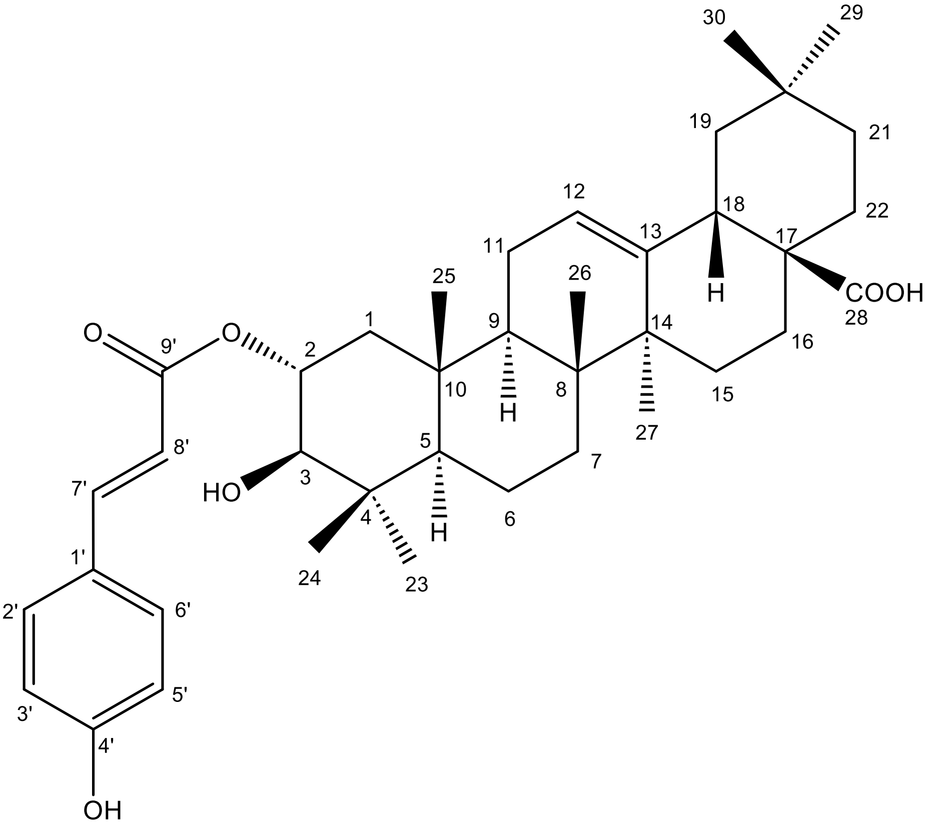

(F2-1) 2-*O*-trans-*p*-coumaroyl-maslinic acid ($C_{39}H_{54}O_6$, 618 g.mol^{-1})

White amorphous powder; ESI-MS (negative-ion mode) *m/z*: 617 [M-H]$^-$; HR-ESI-Orbitrap-MS (negative-ion mode) *m/z*: 617.3863 [M-H]$^-$; (calcd. 617.3837 for $C_{39}H_{53}O_6$ [M-H]$^-$); ^{1}H-NMR spectrum (MeOD ; 500 MHz): δ 7.65 (**CH**, *d*, *J* = 16 Hz, H-7′), 7.48 (**CH**, *d*, *J* = 8.2 Hz, H-2′), 7.48 (**CH**, *d*, *J* = 8.2 Hz, H-6′), 6.82 (**CH**, *d*, *J* = 8.2 Hz; H-3′), 6.82 (**CH**, *d*, *J* = 8.2 Hz; H-5′), 6.36 (**CH**, d, *J* = 16 Hz, H-8′), 5.26 (**CH**, *br.s*, H-12), 5.07 (**CH**, *ddd*, *J* = 11.8, 10.3, 4.6 Hz, H-2), 3.27 (**CH**, *d*, *J* = 10.3 Hz, H-3), 2.87 (**CH**, *dd*, *J* = 14.5, 4.9 Hz, H-18), 2.07 (**CH**$_2$, *m*, H-1β), 2.03 (**CH**$_2$, *m*, H-16β), 1.96 (**CH**$_2$, *m*, H-11β), 1.90 (**CH**$_2$, *m*, H-11α), 1.81 (**CH**$_2$, *m*, H-15β), 1.75 (**CH**$_2$, *m*, H-7β), 1.71 (**CH**$_2$, *m*, H-19β), 1.69 (**CH**, *m*, H-9), 1.63 (**CH**$_2$, *m*, H-6β), 1.63 (**CH**$_2$, *m*, H-16α), 1.55 (**CH**$_2$, *m*, H-22β), 1.55 (**CH**$_2$, *m*, H-7α), 1.50 (**CH**$_2$, *m*, H-6α), 1.41 (**CH**$_2$, *m*, H-21β), 1.37 (**CH**$_2$, m, H-22α), 1.23 (**CH**$_2$, *m*, H-21α), 1.19 (**CH**$_3$, *s*, H-27), 1.15 (**CH**$_2$, *m*, H-19α), 1.12 (**CH**$_3$, *s*, H-25), 1.12 (**CH**$_2$, *m*, H-15α), 1.09 (**CH**$_3$, *s*, H-23), 1.03 (**CH**$_2$, *m*, H-1α), 0.95 (**CH**$_3$, s, H-30), 0.95 (**CH**, *m*, H-5), 0.92 (**CH**$_3$, *s*, H-29), 0.90 (**CH**$_3$, *s*, H-24), 0.85 (**CH**$_3$, *s*, H-26), and ^{13}C-NMR spectrum (MeOD, 125 MHz): 182.16 (C-28), 169.29 (C-9′), 161.20 (C-4′), 146.23 (C-7′), 145.39 (C-13), 131.09 (C-6′), 131.09 (C-2′), 127.30 (C-1′), 123.31 (C-12), 116.81 (C-5′), 116.81 (C-3′), 115.87 (C-8′), 81.10 (C-3), 73.82 (C-2), 56.58 (C-5), 49.36 (C-9), 47.68 (C-17), 47.29 (C-19), 45.18 (C-1), 42.93 (C-8), 42.75 (C-18), 40.98 (C-4), 40.60 (C-14), 39.47 (C-10), 34.91 (C-21), 33.82 (C-7), 33.82 (C-22), 33.58 (C-29), 31.63 (C-20), 29.20 (C-23), 28.82 (C-15), 26.41 (C-27), 24.56 (C-11), 24.07 (C-16), 23.99 (C-30), 19.56 (C-6), 17.70 (C-26), 17.42 (C-24), 16.92 (C-25)

^{1}H spectrum

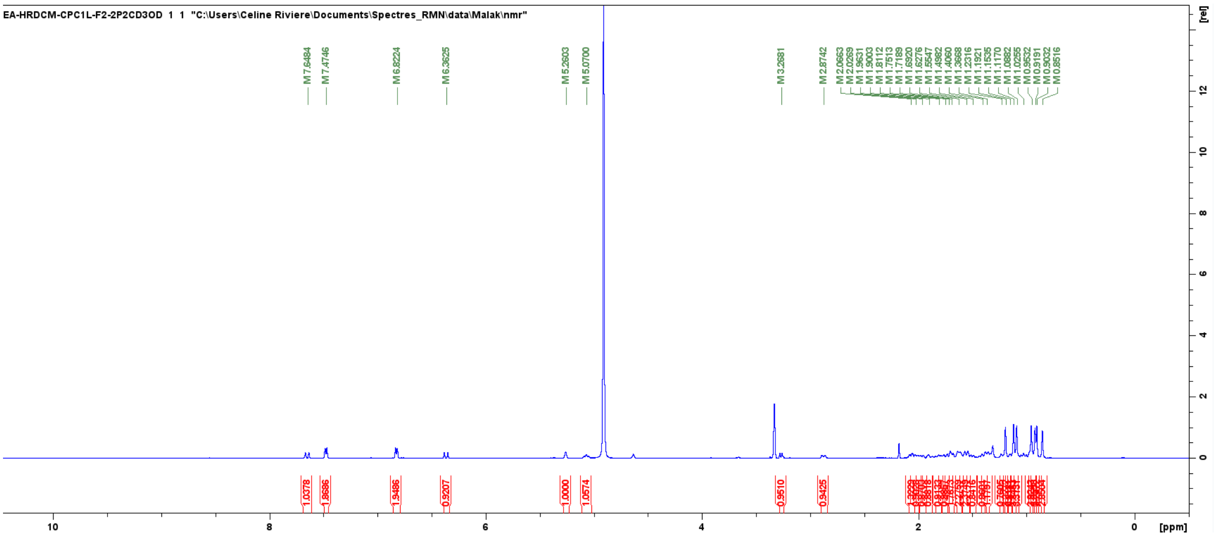

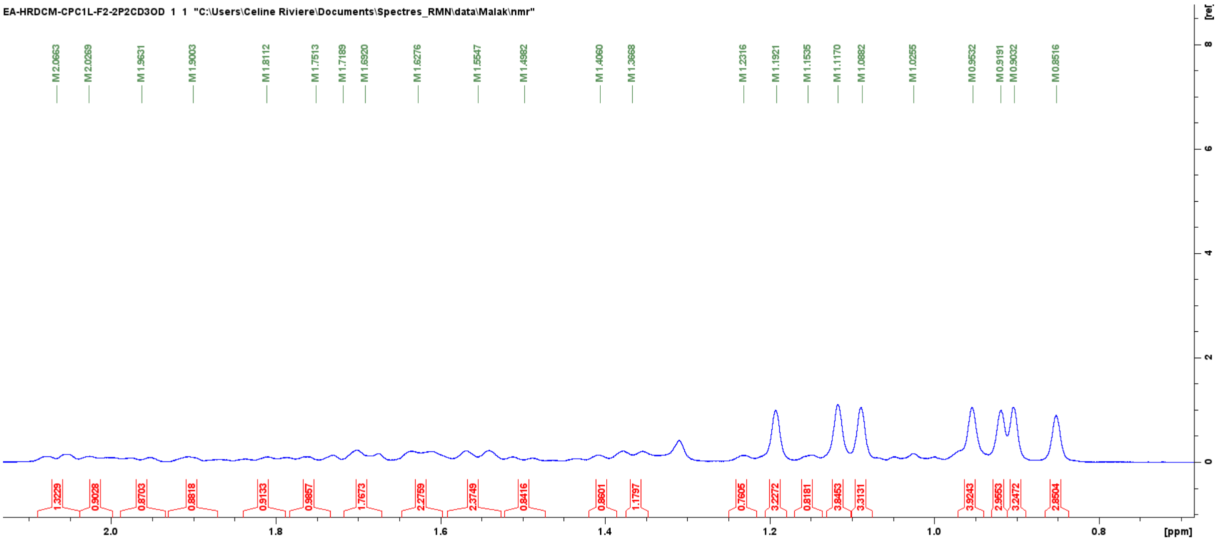

^{13}C spectrum

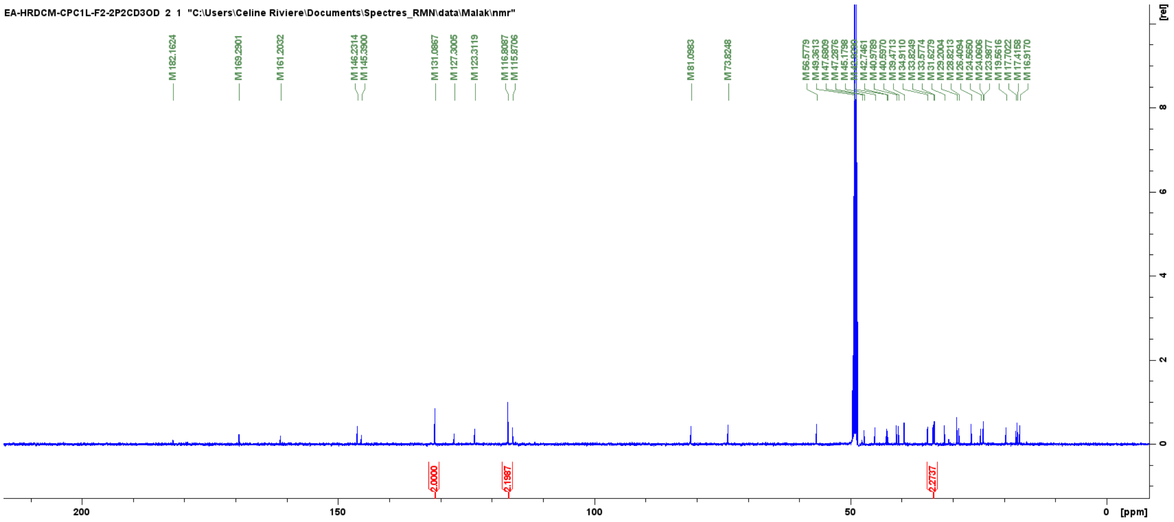

COSY spectrum

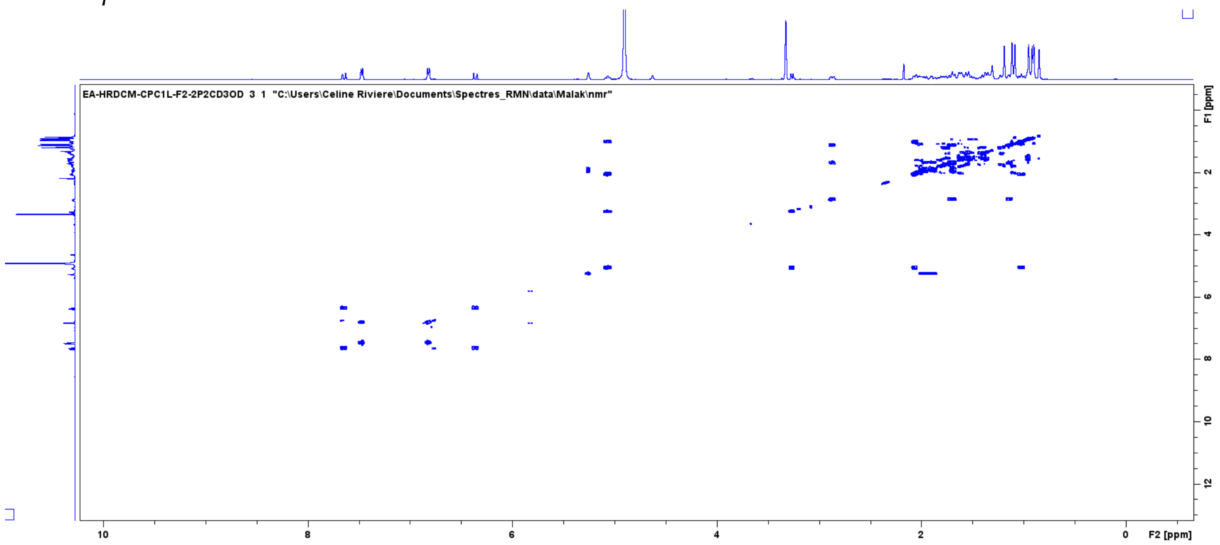

HSQC spectrum

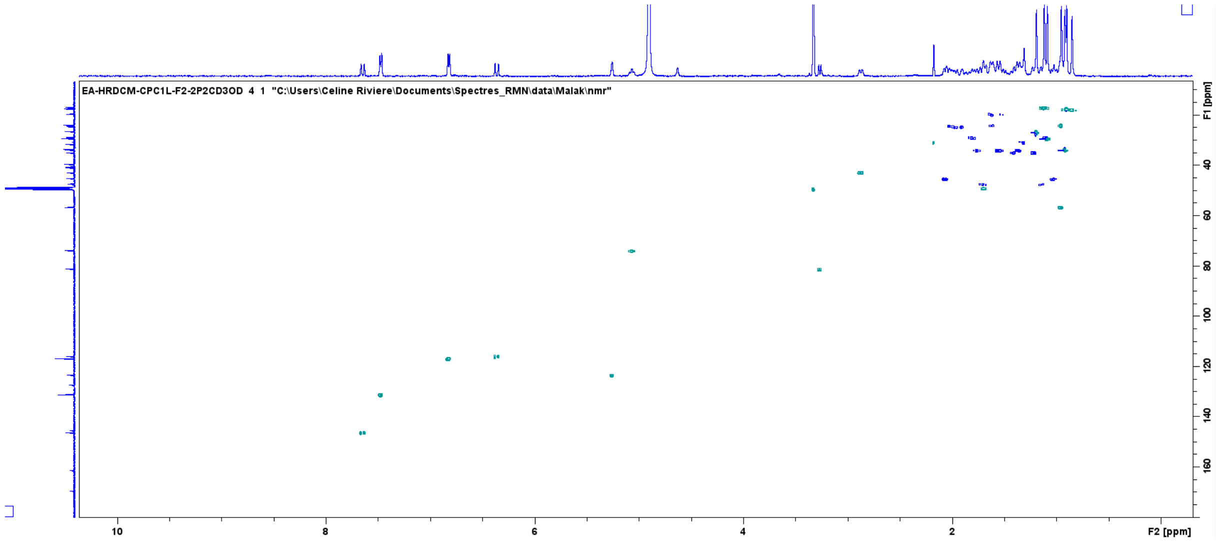

HMBC spectrum

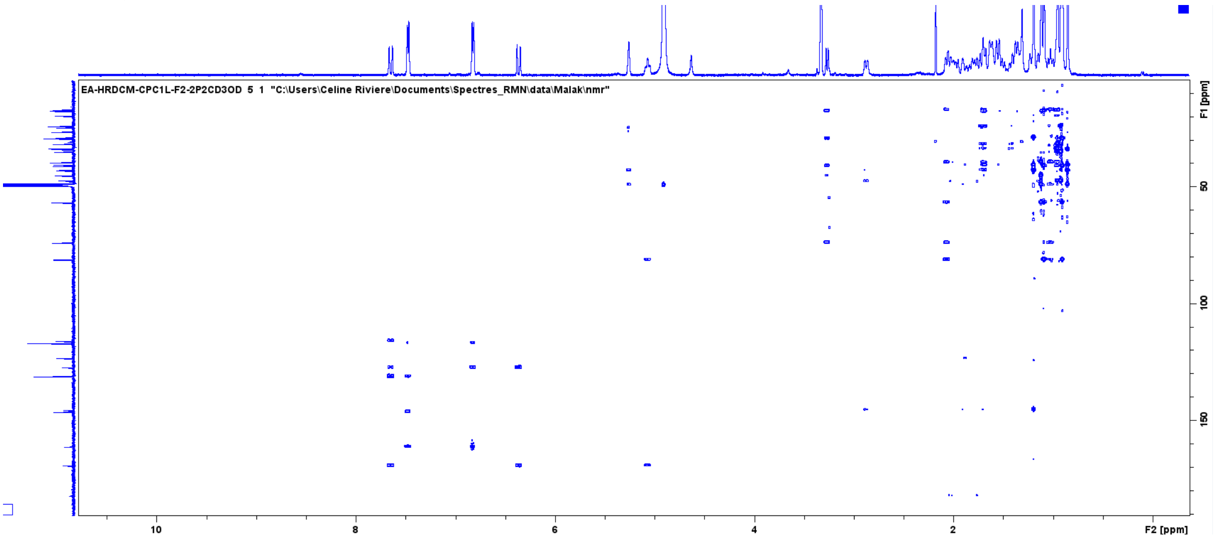

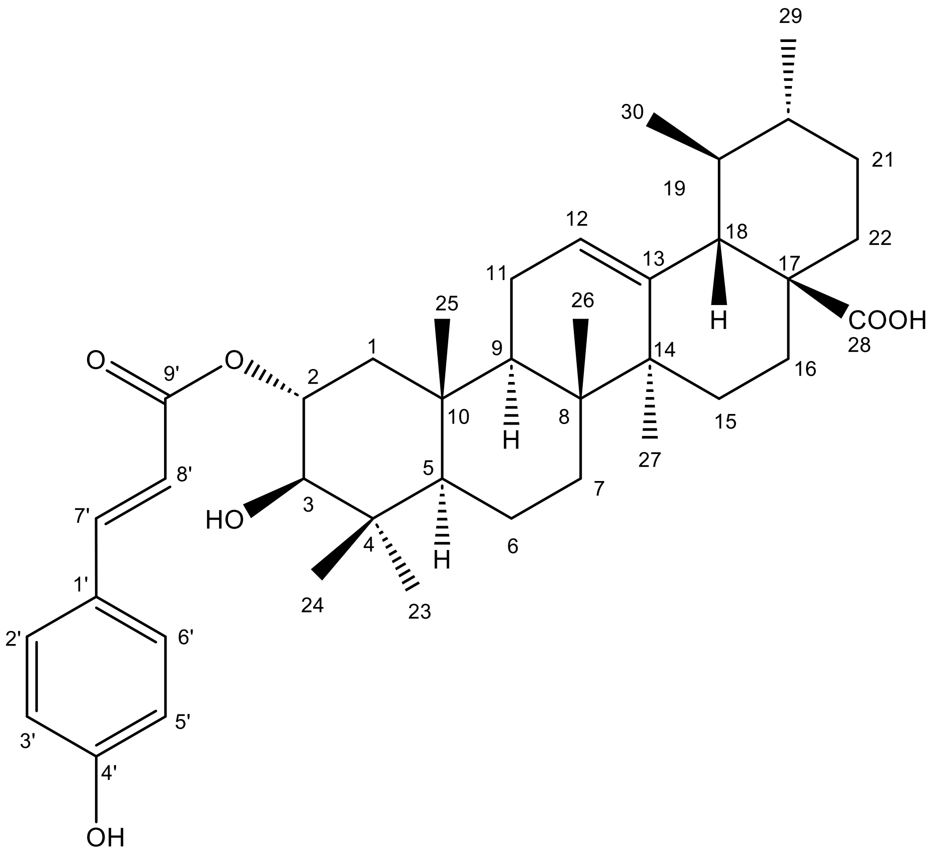

(F2-2) 3β-hydroxy-2*α*-*trans*-*p*-coumaryloxy-urs-12-en-28-oic acid ($C_{39}H_{54}O_6$, 618 g.mol^{-1})

White amorphous powder; ESI-MS (negative-ion mode) *m*/*z*: 617.63 [M-H]$^-$; HR-ESI-Orbitrap-MS (negative-ion mode) *m*/*z*: 617.3848 [M-H]$^-$; (calcd. 617.3837 for $C_{39}H_{53}O_6$ [M-H]$^-$) ; ^{1}H-NMR spectrum (MeOD ; 500 MHz): *δ* 7.65 (C**H**, *d*, *J* = 16 Hz, H-7′), 7.47 (C**H**, *d*, *J* = 8.2 Hz, H-2′), 7.47 (C**H**, d, *J* = 8.2 Hz, H-6′), 6.82 (C**H**, *d*, *J* = 8.2 Hz, H-3′), 6.82 (C**H**, *d*, *J* = 8.2 Hz, H-5′), 6.36 (C**H**, *d*, *J* = 16 Hz, H-8′), 5.24 (C**H**, *br. s*, H-12), 5.08 (C**H**, *ddd*, *J* = 11.56, 10.20, 4.45 Hz , H-2), 3.27 (C**H**, *d*, *J* = 10.20 Hz, H-3), 2.23 (C**H**, *d*, *J* = 11.04 Hz, H-18), 2.09 (C$\mathbf{H_2}$, *m*, H-1β), 2.04 (C$\mathbf{H_2}$, *m*, H-16β), 2.00 (C$\mathbf{H_2}$, *m*, H-15β), 1.96 (C$\mathbf{H_2}$, *m*, H-11), 1.72 (C$\mathbf{H_2}$, *m*, H-22β), 1.66 (C$\mathbf{H_2}$, *m*, H-16*α*), 1.66 (C$\mathbf{H_2}$, *m*, H-22*α*), 1.64 (C**H**, *m*, H-9), 1.61 (C$\mathbf{H_2}$, *m*, H-6β), 1.59 (C$\mathbf{H_2}$, *m*, H-7β), 1.53 (C$\mathbf{H_2}$, *m*, H-21β), 1.50 (C$\mathbf{H_2}$, m, H-6*α*), 1.40 (C$\mathbf{H_2}$, *m*, H-7*α*), 1.39 (C**H**, *m*, H-20), 1.36 (C$\mathbf{H_2}$, *m*, H-21*α*), 1.15 (C$\mathbf{H_3}$, *s*, H-27), 1.13 (C$\mathbf{H_3}$, *s*, H-25), 1.12 (C$\mathbf{H_2}$, *m*, H-15*α*), 1.09 (C$\mathbf{H_3}$, *s*, H-23), 1.05 (C$\mathbf{H_2}$, *m*, H-1*α*), 1.02 (C**H**, *m*, H-19), 0.98 (C$\mathbf{H_3}$, *s*, H-29), 0.95 (C**H**, *m*, H-5), 0.90 (C$\mathbf{H_3}$, *s*, H-24), 0.90 (C$\mathbf{H_3}$, s, H-30), 0.88 (C$\mathbf{H_3}$, *s*, H-26), and ^{13}C-NMR spectrum (MeOD, 125 MHz): 181.12 (C-28), 169.35 (C-9′), 161.22 (C-4′), 146.23 (C-7′), 139.86 (C-13), 131.10 (C-2′), 131.10 (C-6′), 127.31 (C-1′), 126.49 (C-12), 116.80 (C-3′), 116.80 (C-5′), 115.89 (C-8′), 81.11 (C-3), 73.81 (C-2), 56.57 (C-5), 54.38 (C-18), 49.36 (C-17), 49.21 (C-9), 45.35 (C-1), 43.30 (C-8), 40.98 (C-4), 40.83 (C-14), 40.47 (C-20), 40.42 (C-19), 39.41 (C-10), 38.15 (C-22), 34.15 (C-7), 31.81 (C-21), 29.24 (C-15), 29.21 (C-23), 25.34 (C-16), 24.42 (C-11), 24.11 (C-27), 21.60 (C-29), 19.54 (C-6), 17.80 (C-26), 17.70 (C-30), 17.48 (C-24), 17.06 (C-25)

^{1}H spectrum

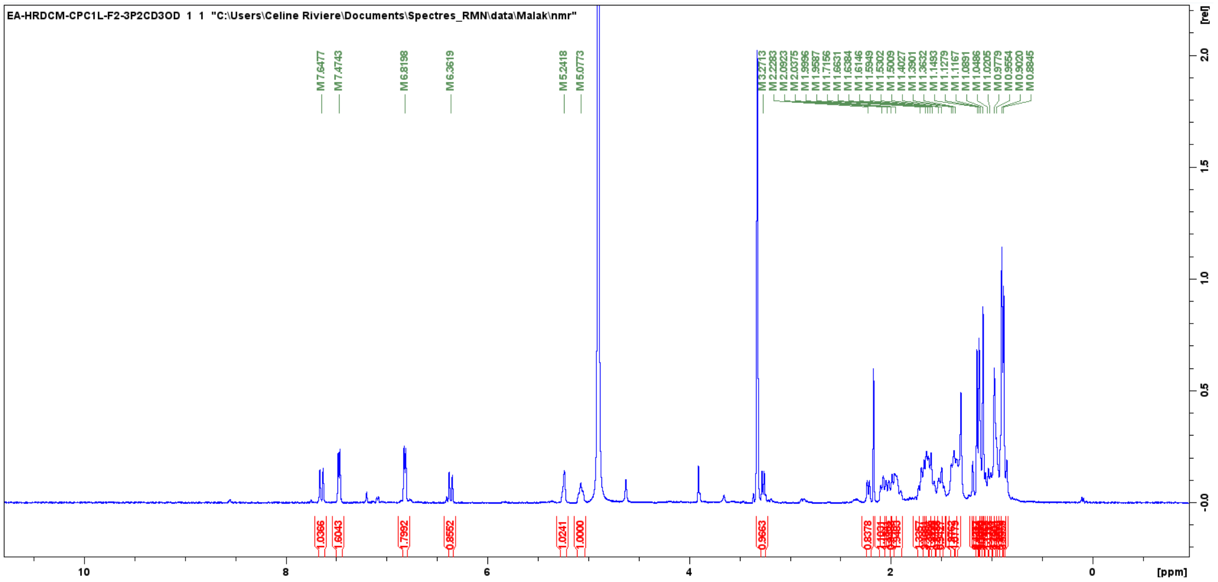

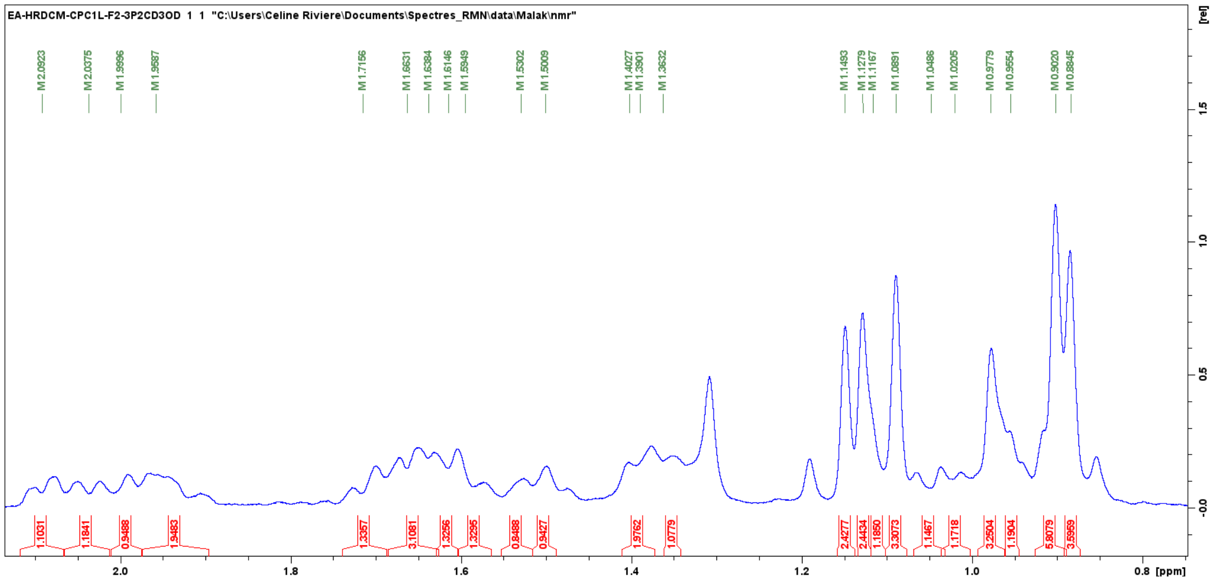

^{13}C spectrum

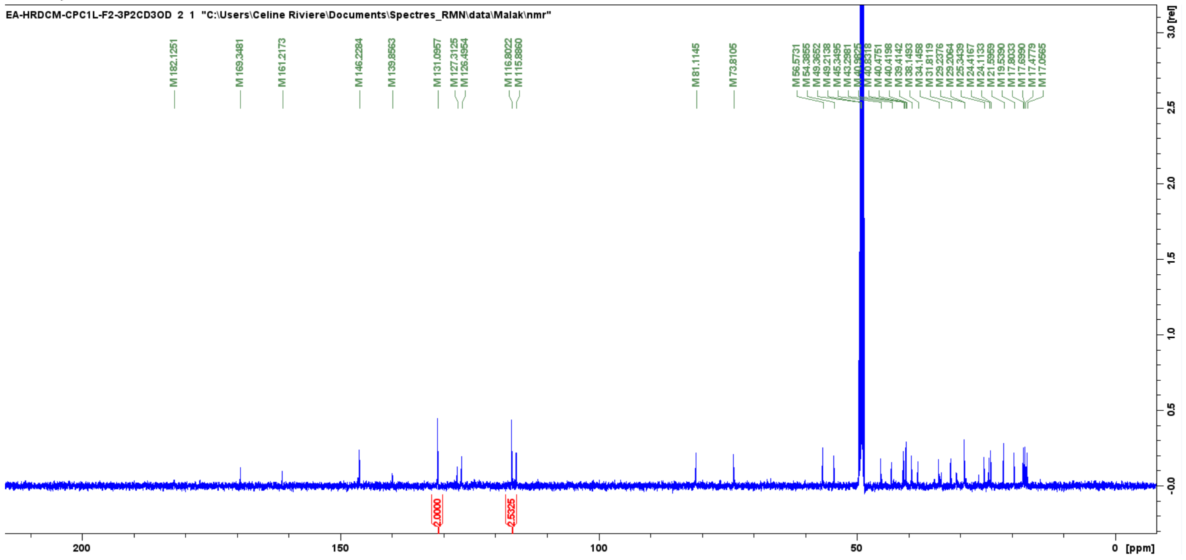

COSY spectrum

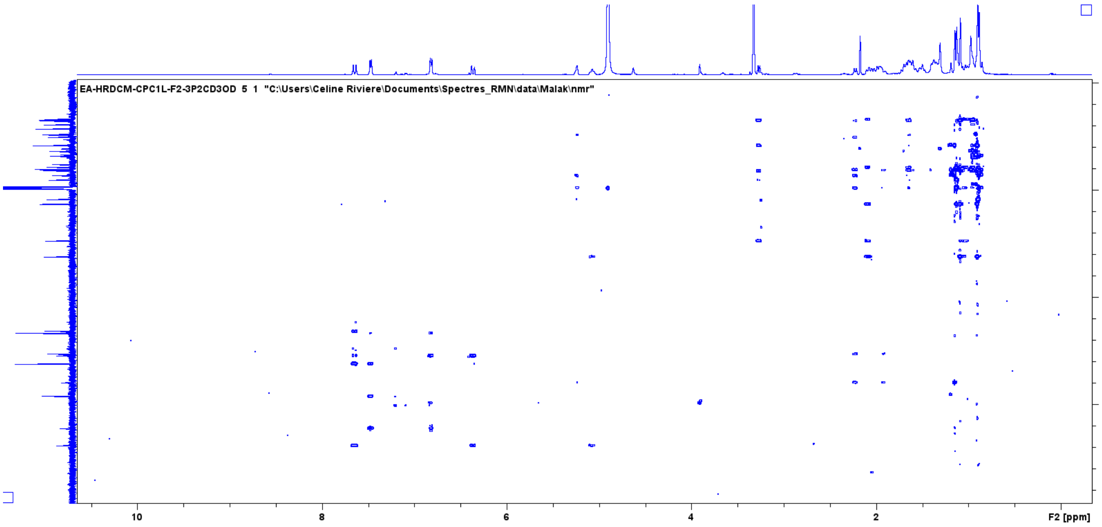

HSCQ spectrum

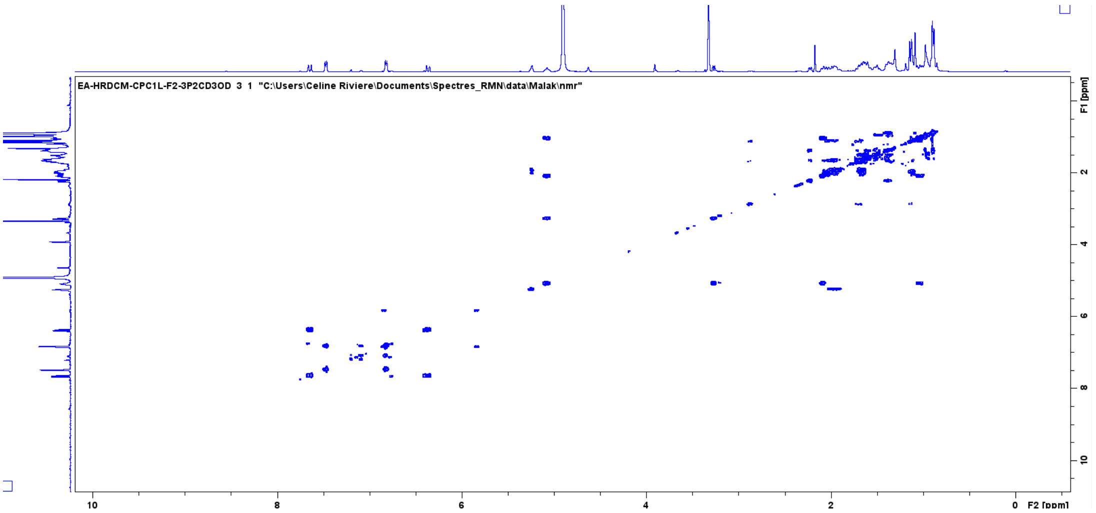

HMBC spectrum

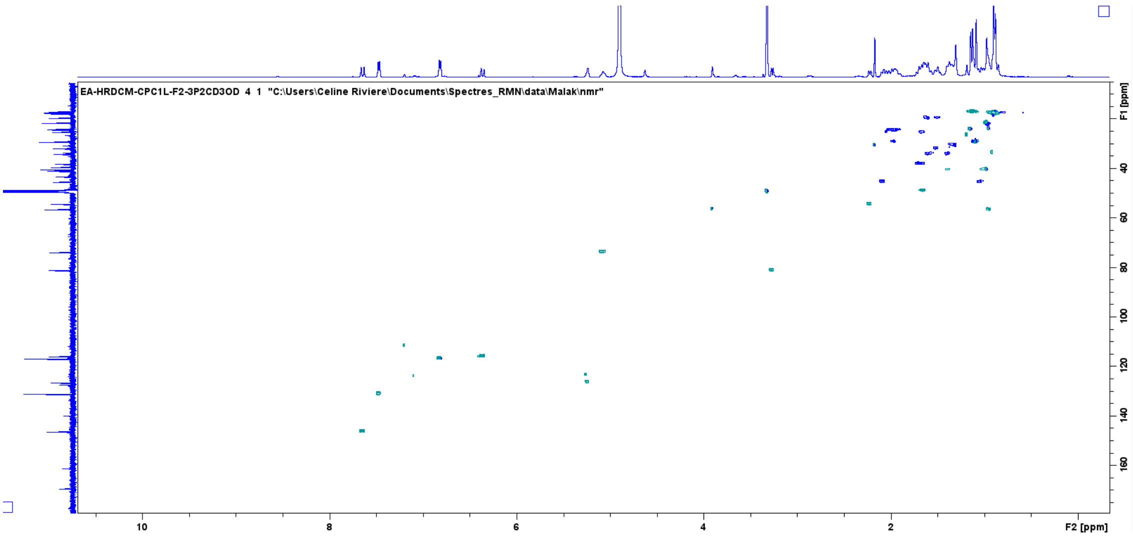

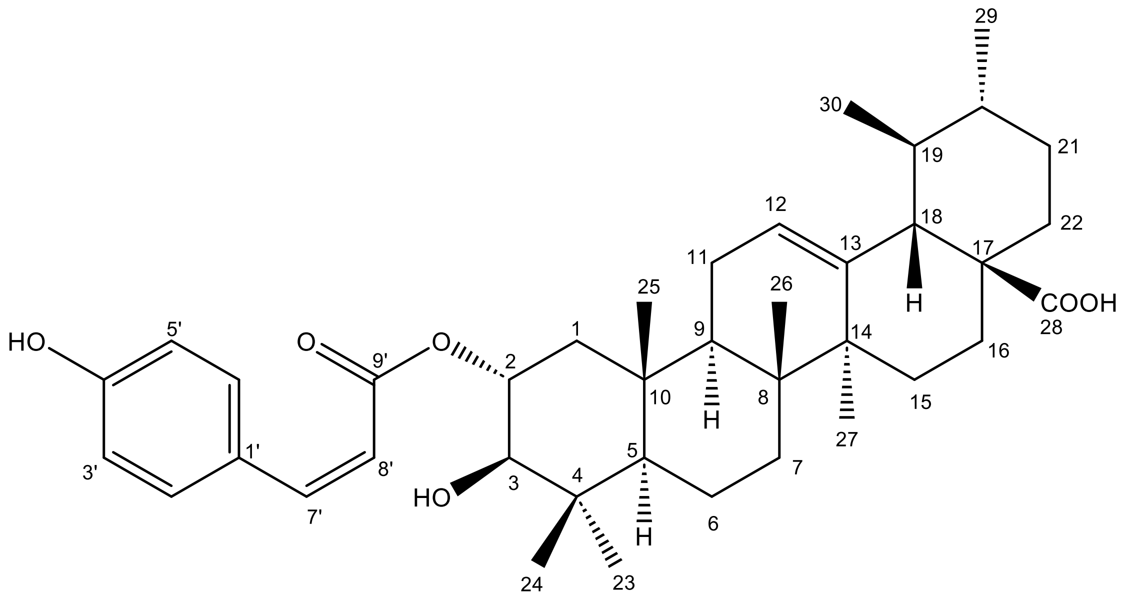

(F2-3) 3β-hydroxy-2α-cis-*p*-coumaryloxy-urs-12-en-28-oic acid ($C_{39}H_{54}O_6$, 618 g.mol^{-1})

White amorphous powder; ESI-MS (negative-ion mode) *m/z*: 617.63 [M-H]$^-$; HR-ESI-Orbitrap-MS (negative-ion mode) *m/z*: 617.3848 [M-H]$^-$; (calcd. 617.3837 for $C_{39}H_{53}O_6$ [M-H]$^-$); **^{1}H-NMR spectrum** (MeOD ; 500 MHz): *δ* 7.67 (C**H**, *d*, *J* = 8.45 Hz, H-2′), 7.67 (C**H**, *d*, *J* = 8.45 Hz, H-6′), 6.86 (C**H**, d, *J* = 12.90 Hz, H-7′), 6.77 (C**H**, *d*, *J* = 8.45 Hz, H-3′), 6.77 (C**H**, *d*, *J* = 8.45 Hz, H-5′), 5.83 (C**H**, d, *J* = 12.90 Hz, H-8′), 5.25 (C**H**, t, *J* = 3.66 Hz, H-12), 5.05 (C**H**, *ddd*, *J* = 11.56, 10.20, 4.45 Hz , H-2), 3.20 (C**H**, *d*, *J* = 10.20 Hz, H-3), 2.23 (C**H**, *d*, *J* = 11.04 Hz, H-18), 2.07 (C**H**$_2$, *m*, H-1β), 2.05 (C**H**$_2$, *m*, H-16β), 1.99 (C**H**$_2$, *m*, H-11), 1.96 (C**H**$_2$, *m*, H-15β), 1.72 (C**H**$_2$, *m*, H-22β), 1.67 (C**H**$_2$, *m*, H-16α), 1.67 (C**H**$_2$, *m*, H-22α), 1.64 (C**H**, *m*, H-9), 1.61 (C**H**$_2$, *m*, H-6β), 1.58 (C**H**$_2$, *m*, H-7β), 1.53 (C**H**$_2$, *m*, H-21), 1.48 (C**H**$_2$, m, H-6α), 1.40 (C**H**, m, H-20), 1.38 (C**H**$_2$, *m*, H-7α), 1.15 (C**H**$_3$, *s*, H-27), 1.12 (C**H**$_3$, *s*, H-25), 1.09 (C**H**$_2$, *m*, H-15α), 1.07 (C**H**$_3$, *s*, H-23), 1.01 (C**H**, *m*, H-19), 0.98 (C**H**$_2$, *m*, H-1α), 0.98 (C**H**$_3$, *s*, H-29), 0.93 (C**H**, *m*, H-5), 0.90 (C**H**$_3$, *s*, H-24), 0.90 (C**H**$_3$, *s*, H-30), 0.88 (C**H**$_3$, *s*, H-26), and **^{13}C-NMR spectrum** (MeOD, 125 MHz): 181.79 (C-28), 169.34 (C-9′), 159.99 (C-4′), 144.47 (C-7′), 139.75 (C-13), 133.65 (C-2′), 133.65 (C-6′), 127.73 (C-1′), 126.61 (C-12), 117.46 (C-8′), 115.79 (C-3′), 115.79 (C-5′), 81.05 (C-3), 73.48 (C-2), 56.50 (C-5), 54.33 (C-18), 48.63 (C-17), 49.18 (C-9), 45.26 (C-1), 43.27 (C-8), 40.98 (C-4), 40.82 (C-14), 40.43 (C-20), 40.42 (C-19), 39.39 (C-10), 38.12 (C-22), 34.11 (C-7), 31.77 (C-21), 29.20 (C-15), 29.18 (C-23), 25.31 (C-16), 24.41 (C-11), 24.10 (C-27), 21.57 (C-29), 19.53 (C-6), 17.76 (C-26), 17.69 (C-30), 17.48 (C-24), 17.06 (C-25)

^{1}H spectrum

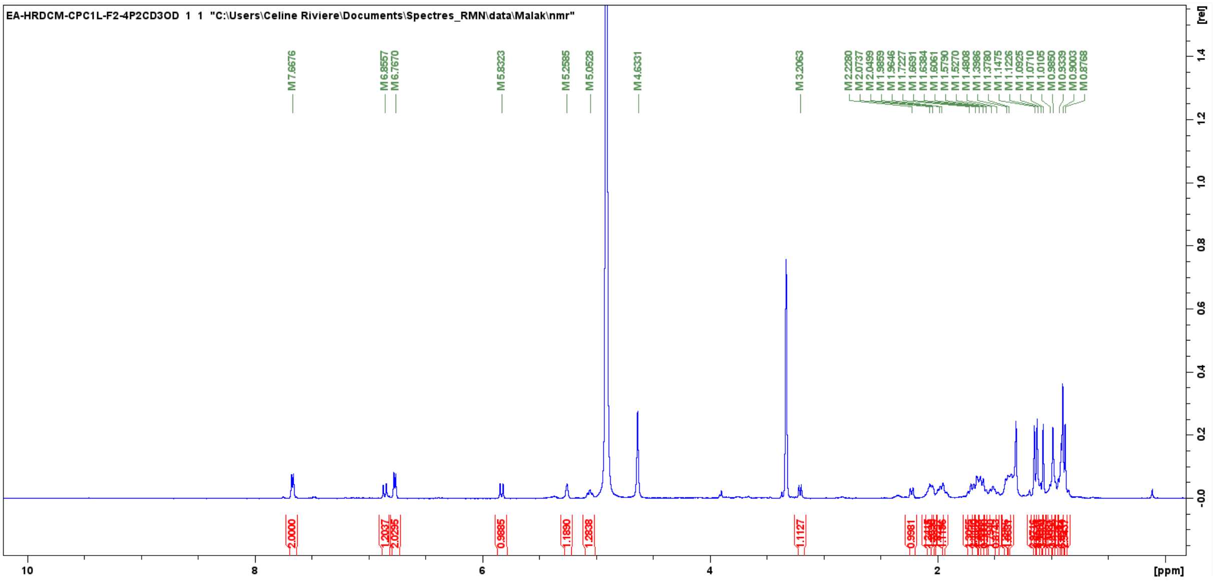

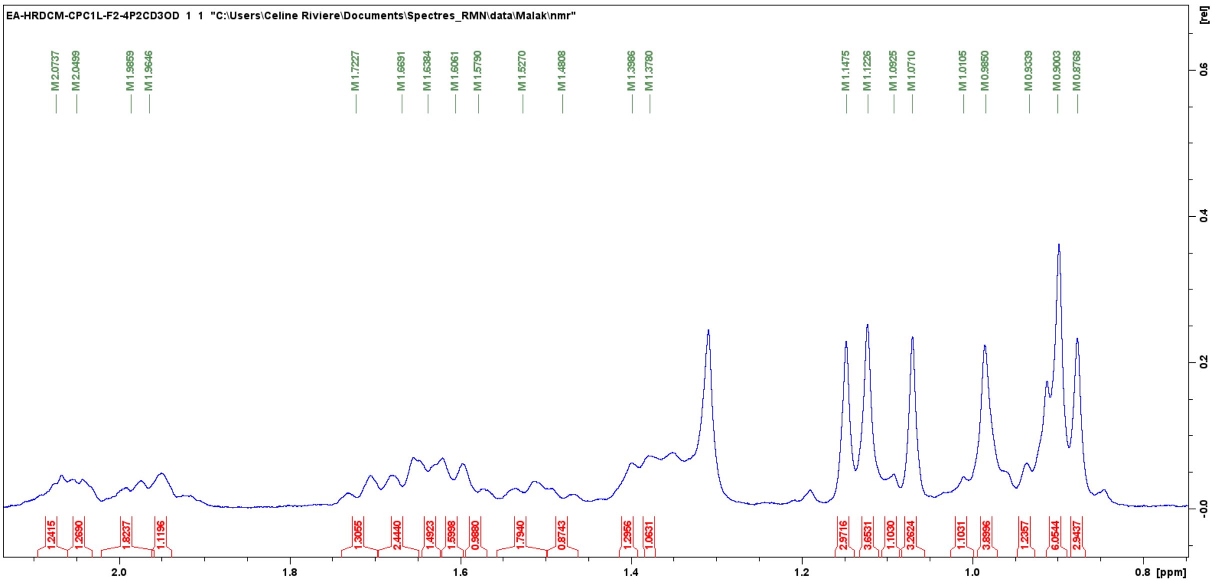

^{13}C spectrum

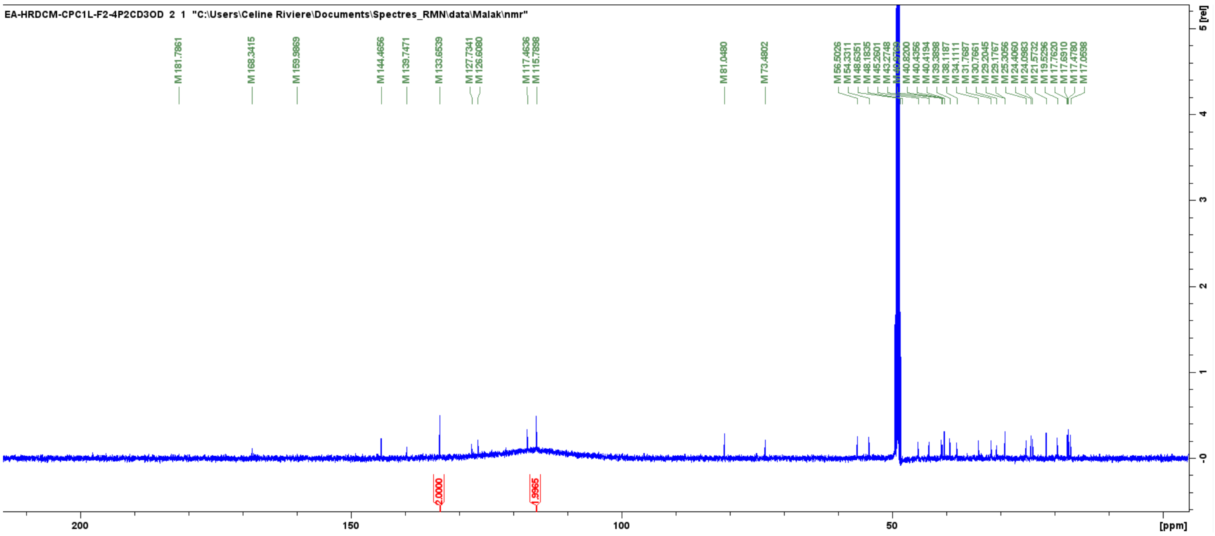

COSY spectrum

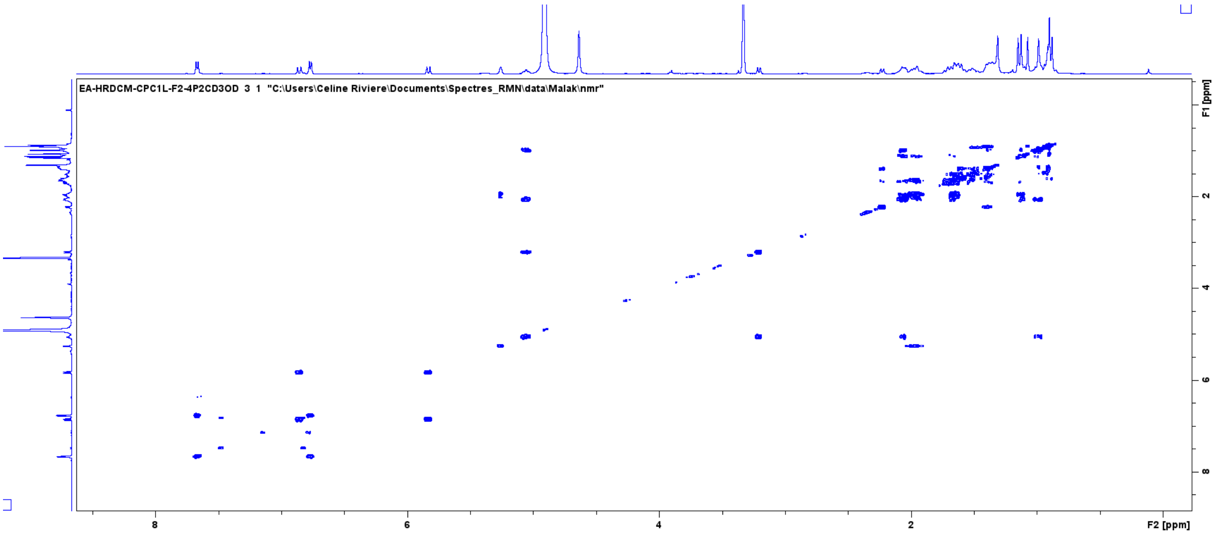

HSQC spectrum

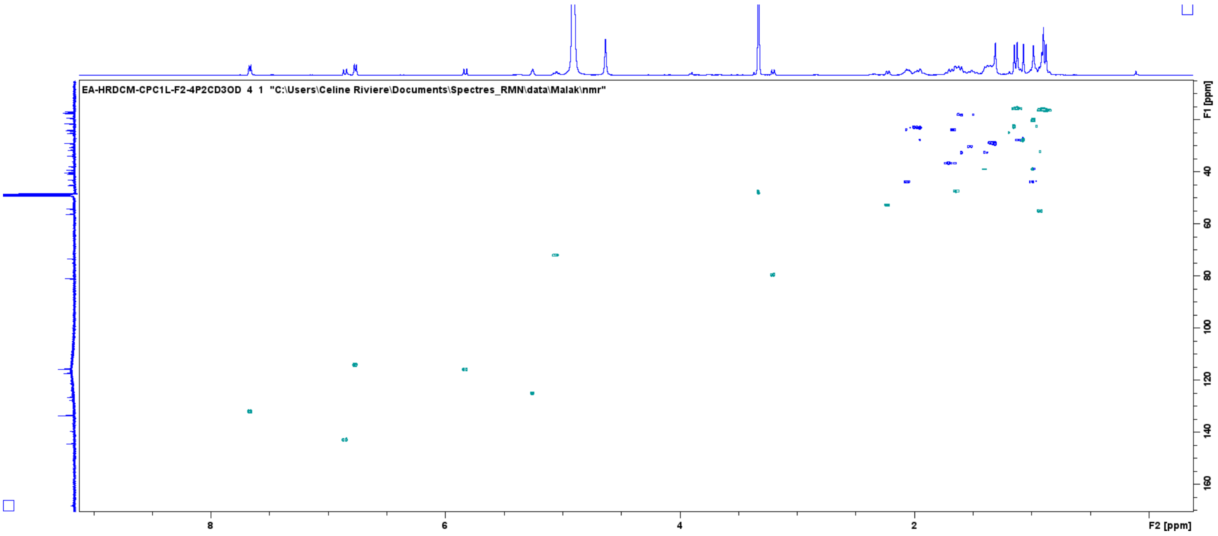

HMBC spectrum

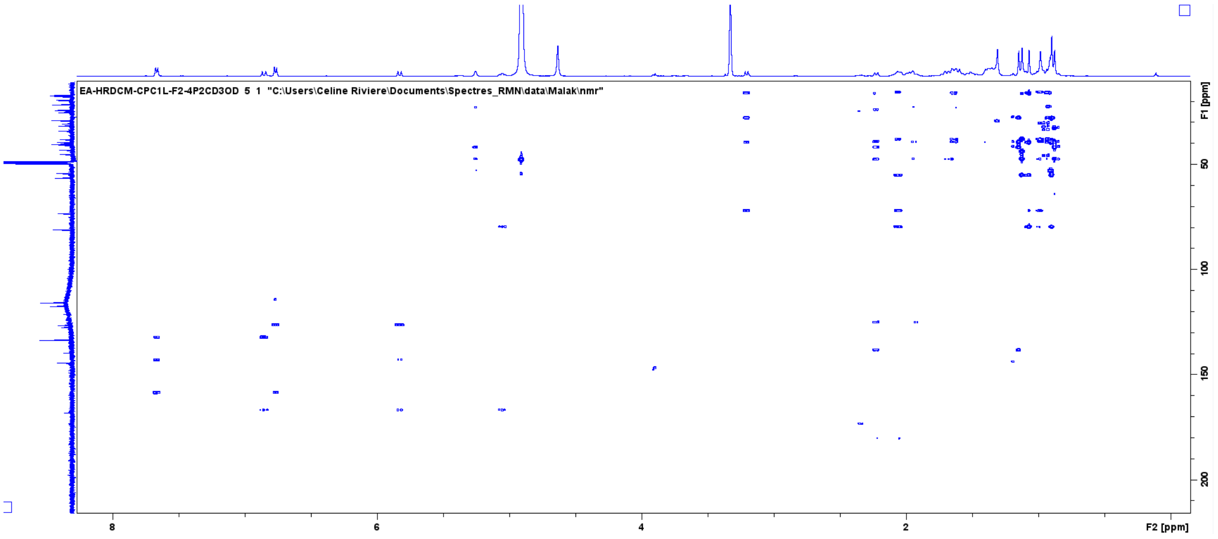

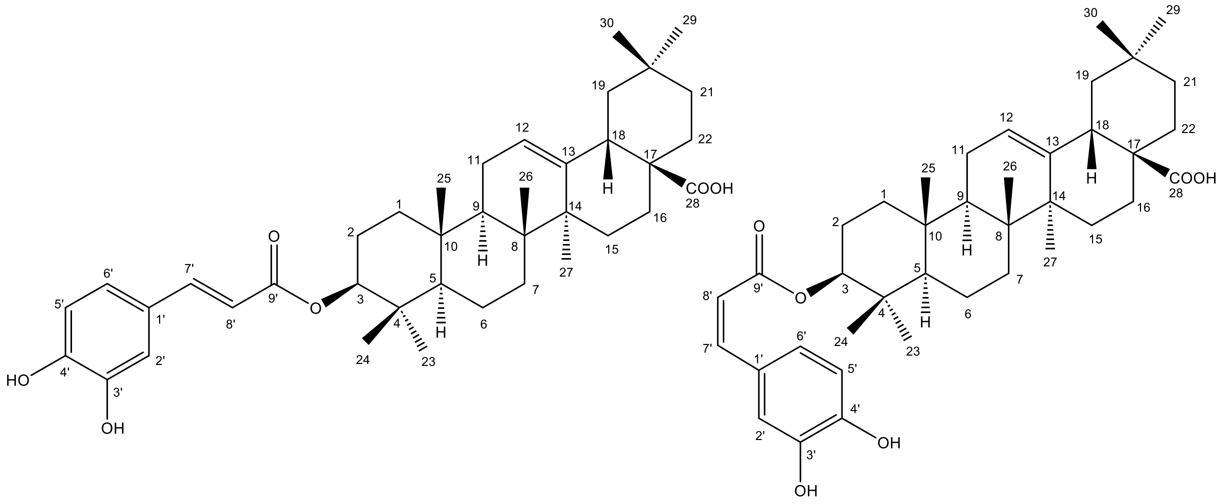

(F4-1) Mixture 3-*O*-*trans*-caffeoyl oleanolic acid / 3-*O*-*cis*-caffeoyl oleanolic acid (70/30) ($C_{39}H_{54}O_6$, 618 g.mol^{-1})

White amorphous powder; **ESI-MS** (negative-ion mode) *m/z*: 617 [M-H]$^-$; **HR-ESI-Orbitrap-MS** (negative-ion mode) *m/z*: 617.3841 [M-H]$^-$; (calcd. 617.3837 for $C_{39}H_{53}O_6$ [M-H]$^-$); **^{1}H-NMR spectrum of 3-*O*-*cis*-caffeoyl oleanolic acid** (MeOD ; 500 MHz): *δ* 7.32 (**CH**, *d*, *J* = 2.08 Hz, H-2′), 7.02 (**CH**, *dd*, *J* = 8.18, 2.08 Hz; H-6′), 6.82 (**CH**, d, *J* = 12.6 Hz, H-7′), 6.74 (**CH**, *d*, *J* = 8.18 Hz, H-5′), 5.76 (**CH**, d, *J* = 12.6 Hz, H-8′), 5.27 (**CH**, t, *J* = 3.66 Hz, H-12), 4.59 (**CH**, *dd*, *J* = 11.72, 4.76 Hz, H-3), 2.88 (**CH**, *dd*, *J* = 14.04, 4.59 Hz , H-18), 2.04 (CH_2, *m*, H-16β), 1.95 (**CH**, *m*, H-9), 1.94 (CH_2, *m*, H-11), 1.82 (CH_2, *m*, H-15β), 1.80 (CH_2, *m*, H-22β), 1.77 (CH_2, *m*, H-2β), 1.73 (CH_2, m, H-19β), 1.72 (CH_2, *m*, H-1β), 1.71 (CH_2, *m*, H-2α), 1.62 (CH_2, *m*, H-16α), 1.60 (CH_2, *m*, H-6β), 1.57 (CH_2, *m*, H-7β), 1.55 (CH_2, *m*, H-22α), 1.49 (CH_2, m, H-6α), 1.42 (CH_2, *m*, H-21β), 1.36 (CH_2, *m*, H-7α), 1.24 (CH_2, m, H-21α), 1.21 (CH_3, *s*, H-27), 1.16 (CH_2, *m*, H-19α), 1.12 (CH_2, *m*, H-1α), 1.09 (CH_2, *m*, H-15α), 1.03 (CH_3, *s*, H-25), 0.99 (CH_3, *s*, H-26), 0.97 (CH_3, *s*, H-30), 0.93 (**CH**, *m*, H-5), 0.93 (CH_3, *s*, H-23), 0.93 (CH_3, *s*, H-29), 0.86 (CH_3, *s*, H-24),

Comparison of ^{1}H spectrum of 3-O-trans-caffeoyl oleanolic acid (red spectrum) and mixture 3-O-trans-caffeoyl oleanolic acid / 3-O-cis-caffeoyl oleanolic acid (70/30) (blue spectrum)

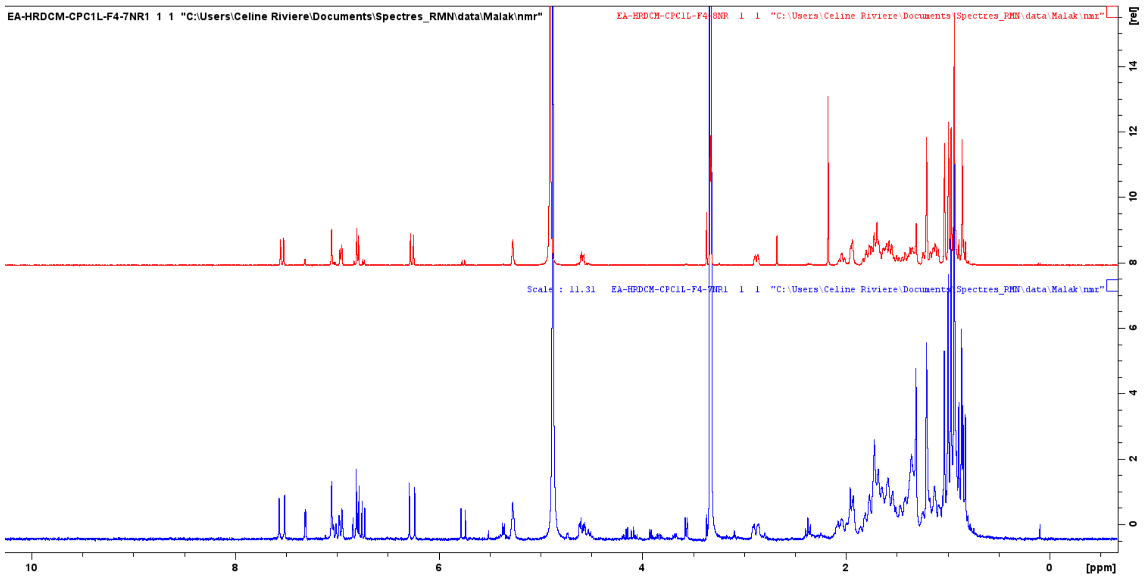

^{1}H *spectrum* of mixture 3-*O*-*trans*-caffeoyl oleanolic acid / 3-*O*-*cis*-caffeoyl oleanolic acid (70/30) (peak picking of protons belonging to the cis-caffeoyl moiety and integration).

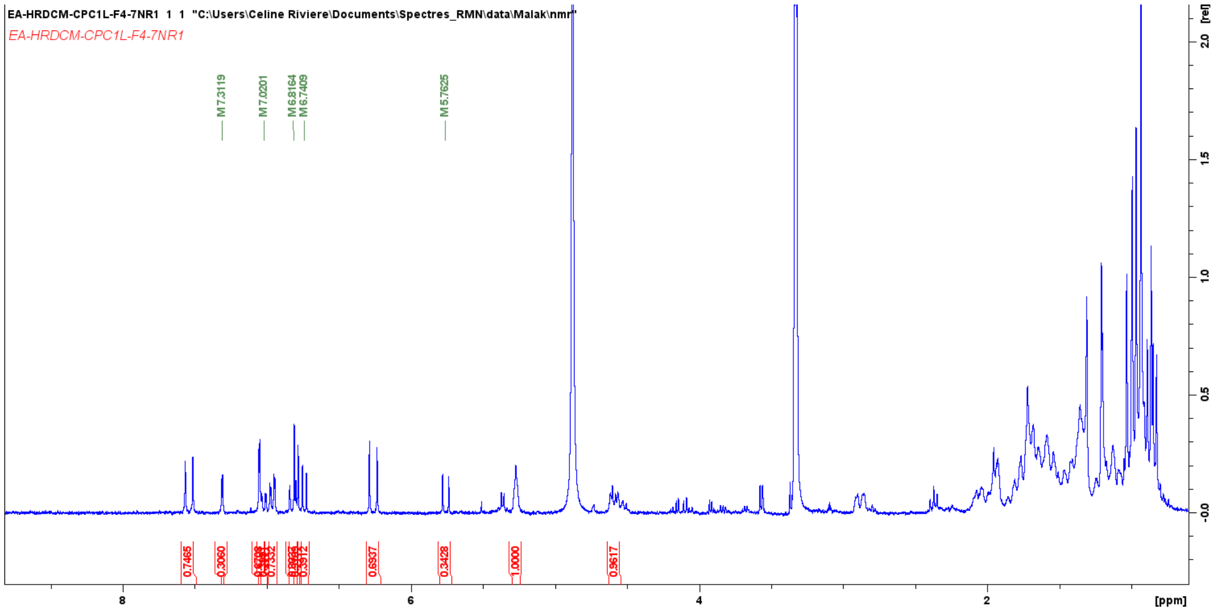

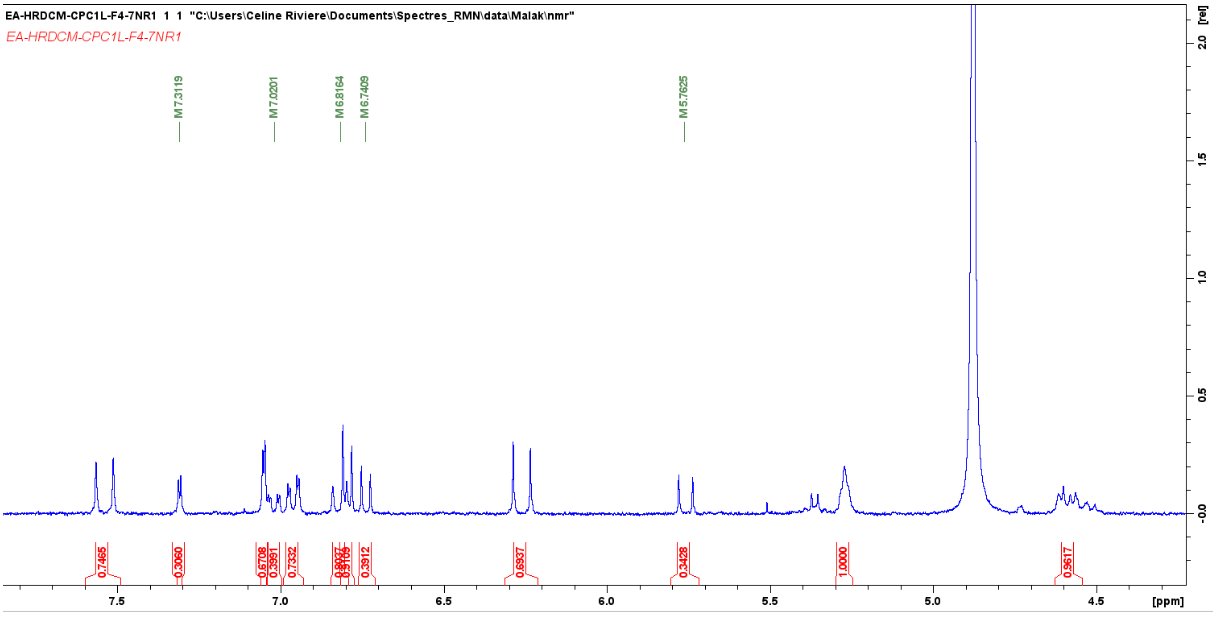

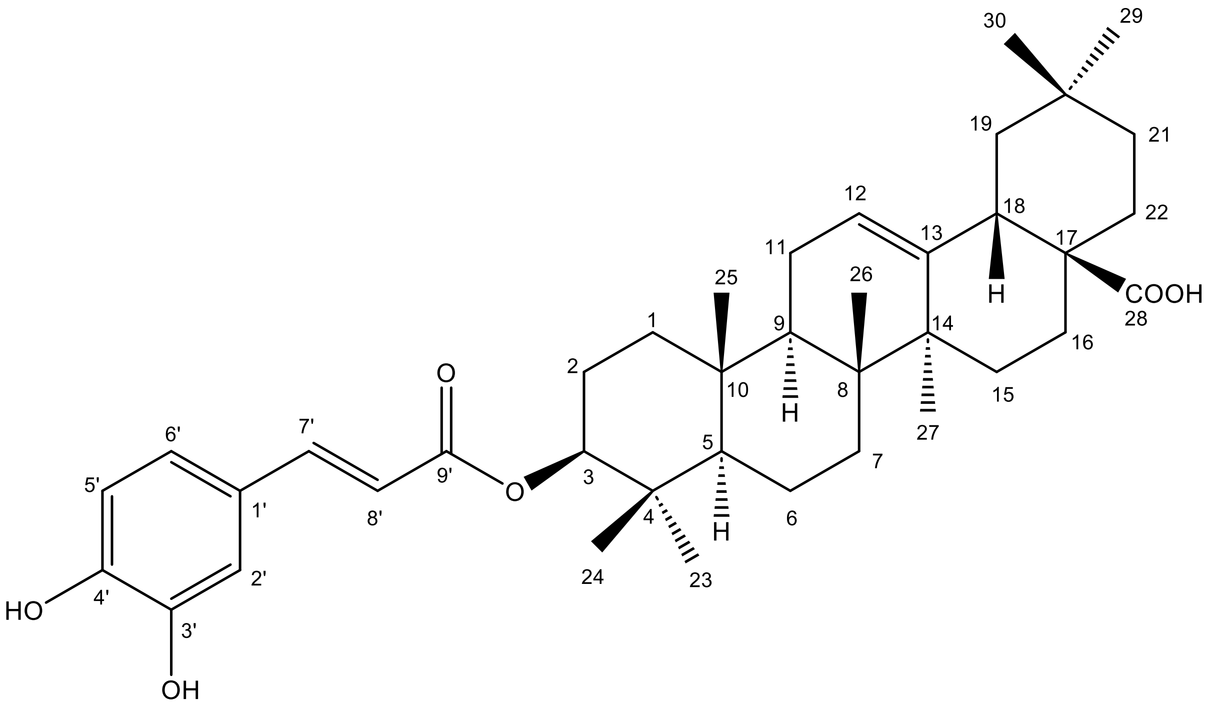

(F4-2) Oleanolic acid caffeate = 3-*O*-*trans*-caffeoyl oleanolic acid ($C_{39}H_{54}O_6$, 618 g.mol^{-1})

White amorphous powder; **ESI-MS** (negative-ion mode) *m/z*: 617 [M-H]$^-$; **HR-ESI-Orbitrap-MS** (negative-ion mode) *m/z*: 617.3841 [M-H]$^-$; (calcd. 617.3837 for $C_{39}H_{53}O_6$ [M-H]$^-$) ; **^{1}H-NMR spectrum** (MeOD ; 500 MHz): *δ* 7.54 (**CH**, d, *J* = 15.9 Hz, H-7′), 7.05 (**CH**, *d*, *J* = 2.08 Hz, H-2′), 6.96 (**CH**, *dd*, *J* = 8.18, 2.08 Hz; H-6′), 6.80 (**CH**, *d*, *J* = 8.18 Hz, H-5′), 6.26 (**CH**, d, *J* = 15.9 Hz, H-8′), 5.27 (**CH**, t, *J* = 3.66 Hz, H-12), 4.59 (**CH**, *dd*, *J* = 11.72, 4.76 Hz, H-3), 2.88 (**CH**, *dd*, *J* = 14.04, 4.59 Hz , H-18), 2.04 (**CH$_2$**, *m*, H-16β), 1.95 (**CH**, *m*, H-9), 1.94 (**CH$_2$**, *m*, H-11), 1.82 (**CH$_2$**, *m*, H-15β), 1.80 (**CH$_2$**, *m*, H-22β), 1.77 (**CH$_2$**, *m*, H-2β), 1.73 (**CH$_2$**, m, H-19β), 1.72 (**CH$_2$**, *m*, H-1β), 1.71 (**CH$_2$**, *m*, H-2*α*), 1.62 (**CH$_2$**, *m*, H-16*α*), 1.60 (**CH$_2$**, *m*, H-6β), 1.57 (**CH$_2$**, *m*, H-7β), 1.55 (**CH$_2$**, *m*, H-22*α*), 1.49 (**CH$_2$**, m, H-6*α*), 1.42 (**CH$_2$**, *m*, H-21β), 1.36 (**CH$_2$**, *m*, H-7*α*), 1.24 (**CH$_2$**, m, H-21*α*), 1.21 (**CH$_3$**, *s*, H-27), 1.16 (**CH$_2$**, *m*, H-19*α*), 1.12 (**CH$_2$**, *m*, H-1*α*), 1.09 (**CH$_2$**, *m*, H-15*α*), 1.03 (**CH$_3$**, *s*, H-25), 0.99 (**CH$_3$**, *s*, H-26), 0.97 (**CH$_3$**, *s*, H-30), 0.93 (**CH**, *m*, H-5), 0.93 (**CH$_3$**, *s*, H-23), 0.93 (**CH$_3$**, *s*, H-29), 0.86 (**CH$_3$**, *s*, H-24), and **^{13}C-NMR spectrum** (MeOD, 125 MHz): 182.04 (C-28), 169.20 (C-9′), 149.57 (C-4′), 146.84 (C-3′), 146.69 (C-7′), 145.28 (C-13), 127.74 (C-1′), 123.53 (C-12), 122.95 (C-6′), 116.50 (C-5′), 115.57 (C-8′), 115.07 (C-2′), 82.28 (C-3), 56.82 (C-5), 49.19 (C-9), 47.66 (C-17), 47.26 (C-19), 42.92 (C-14), 42.74 (C-18), 40.58 (C-8), 39.35 (C-1), 38.99 (C-4), 38.16 (C-10), 34.91 (C-21), 33.91 (C-7), 33.84 (C-22), 33.58 (C-29), 31.63 (C-20), 28.85 (C-15), 28.67 (C-23), 26.41 (C-27), 24.70 (C-2), 24.53 (C-11), 24.07 (C-30), 23.99 (C-16), 19.37 (C-6), 17.73 (C-26), 17.36 (C-24), 15.94 (C-25)

^{1}H spectrum

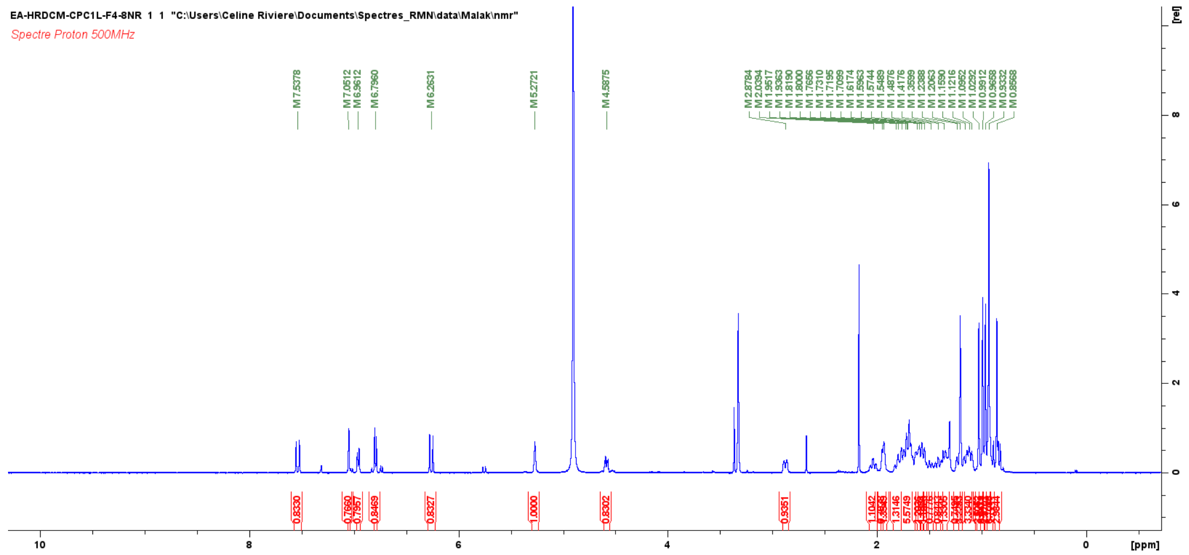

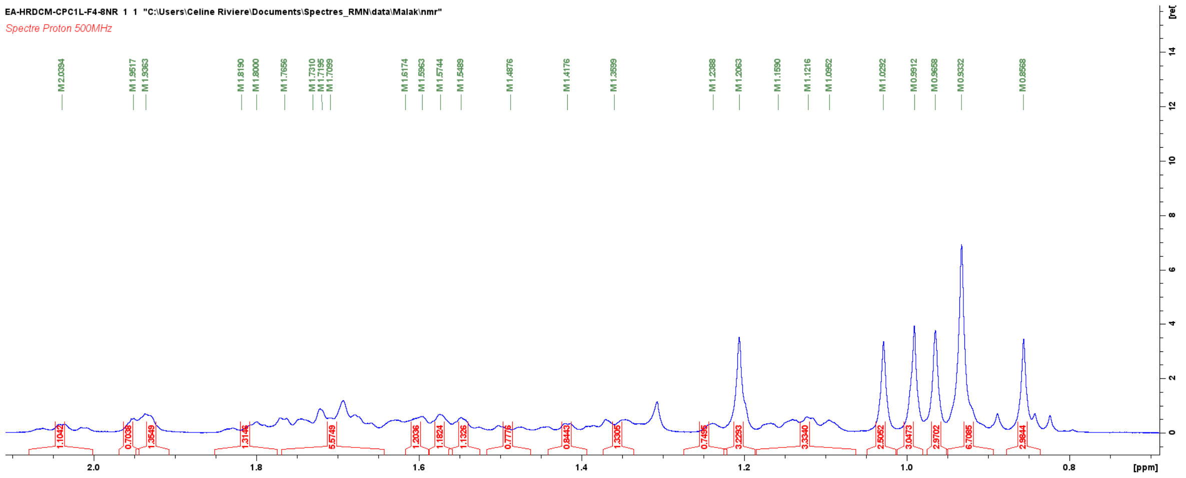

^{13}C spectrum

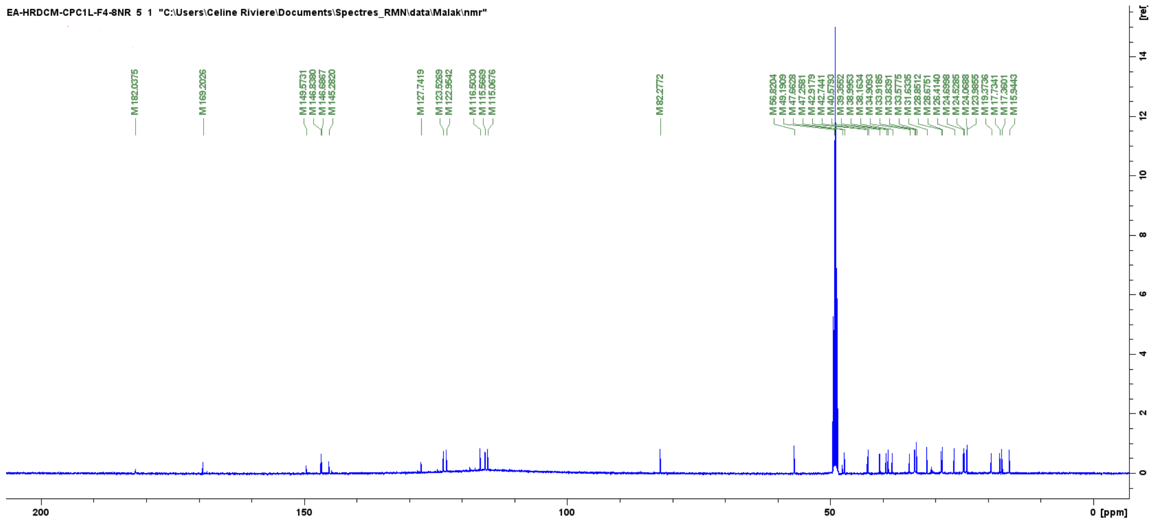

COSY spectrum

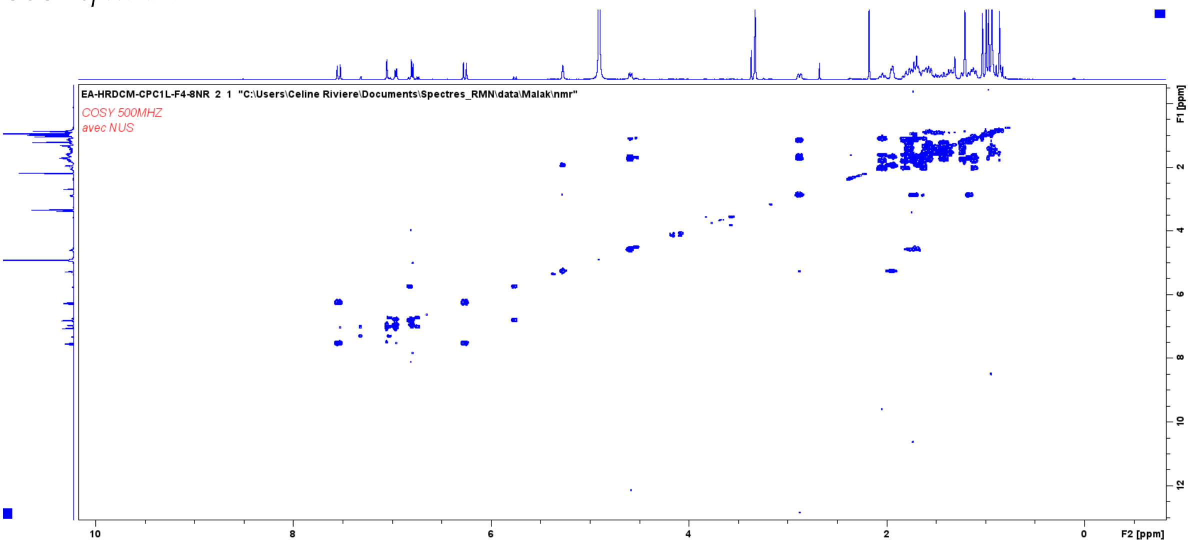

HSQC spectrum

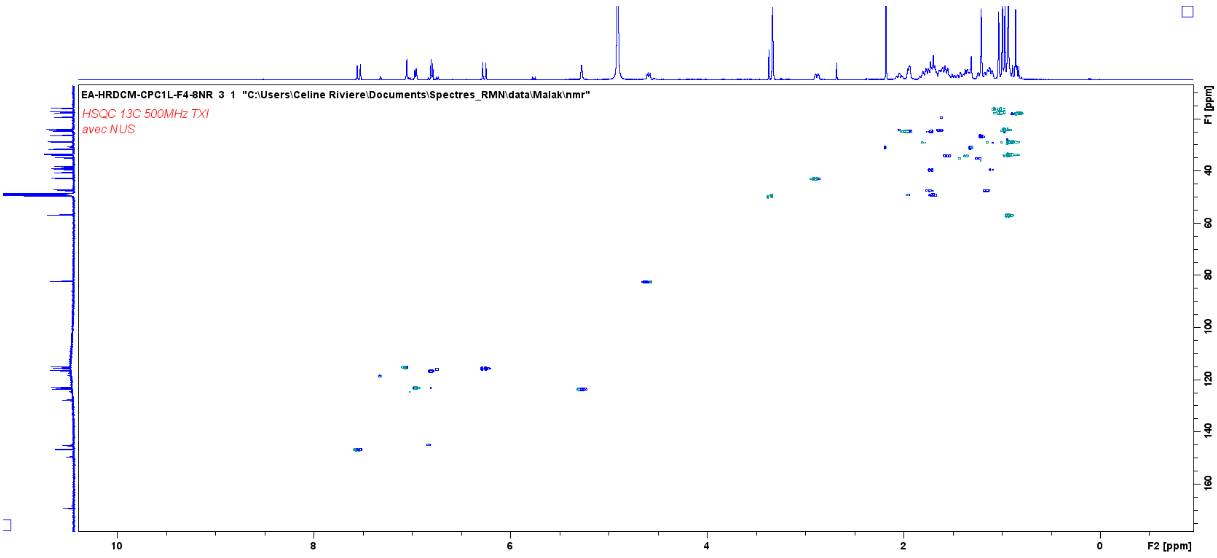

HMBC spectrum

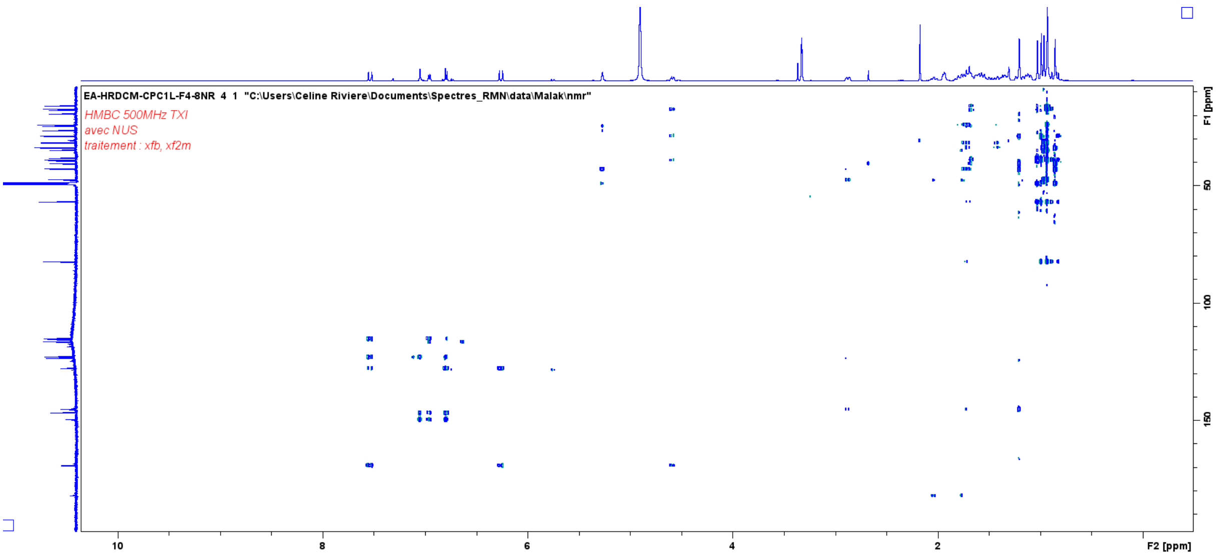

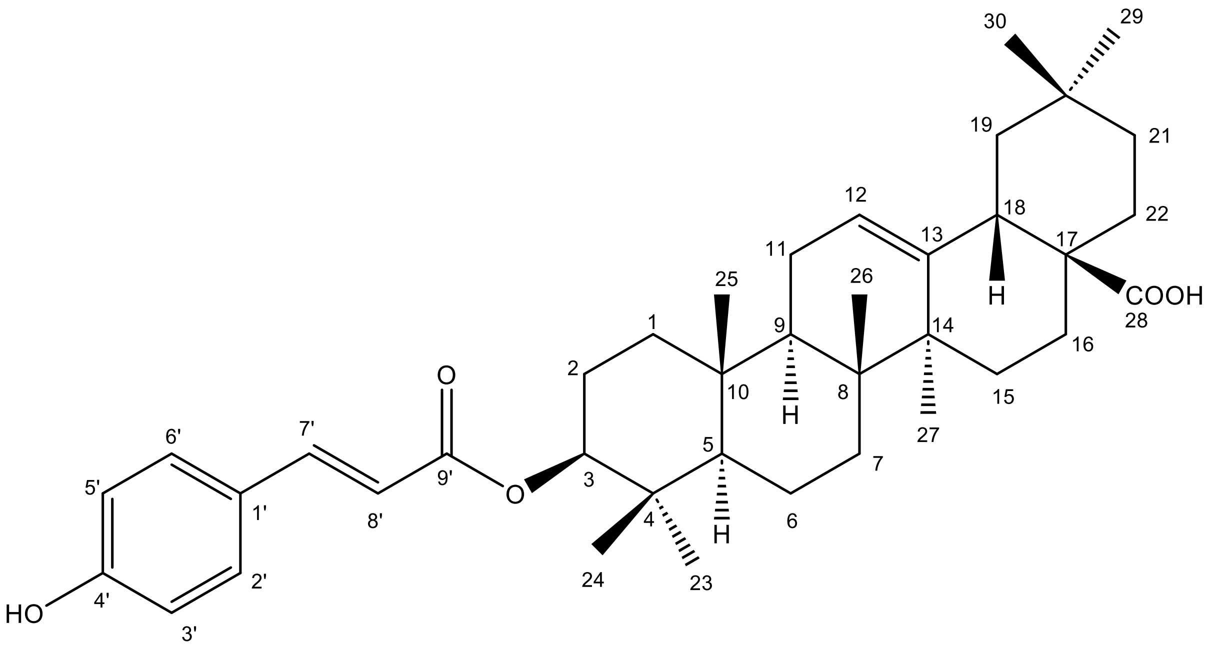

(F7-1) 3-O-*trans*-*p*-coumaroyl oleanolic acid ($C_{39}H_{54}O_5$, 602 g.mol^{-1})

White amorphous powder; ESI-MS (negative-ion mode) *m/z*: 601.72 [M-H]$^-$; HR-ESI-Orbitrap-MS (negative-ion mode) *m/z*: 601.3899 [M-H]$^-$; (calcd. 601.3888 for $C_{39}H_{53}O_5$ [M-H]$^-$); **^{1}H-NMR spectrum** (MeOD ; 500 MHz): δ 7.61 (**C**H, d, *J* = 15.91 Hz, H-7′), 7.48 (**C**H, *d*, *J* = 8.68 Hz, H-2′), 7.48 (**C**H, *d*, *J* = 8.68 Hz, H-6′), 6.83 (**C**H, *d*, *J* = 8.68 Hz, H-3′), 6.83 (**C**H, *d*, *J* = 8.68 Hz, H-5′), 6.33 (**C**H, d, *J* = 15.91 Hz, H-8′), 5.27 (**C**H, t, *J* = 3.59 Hz, H-12), 4.59 (**C**H, *dd*, *J* = 11.72, 4.67 Hz, H-3), 2.88 (**C**H, *dd*, *J* = 14.10, 4.59 Hz, H-18), 2.02 (**C**H_2, *m*, H-16β), 1.94 (**C**H, *m*, H-9), 1.93 (**C**H_2, *m*, H-11β), 1.80 (**C**H_2, *m*, H-15β), 1.77 (**C**H_2, *m*, H-22β), 1.75 (**C**H_3, *m*, H-2β), 1.72 (**C**H_2, m, H-19β), 1.70 (**C**H_2, *m*, H-1β), 1.70 (**C**H_3, *m*, H-2α), 1.68 (**C**H_2, *m*, H-11α), 1.63 (**C**H_2, *m*, H-16α), 1.60 (**C**H_2, *m*, H-6β), 1.58 (**C**H_2, *m*, H-7β), 1.55 (**C**H_2, *m*, H-22α), 1.49 (**C**H_2, m, H-6α), 1.42 (**C**H_2, *m*, H-21β), 1.35 (**C**H_2, *m*, H-7α), 1.24 (**C**H_2, m, H-21α), 1.21 (**C**H_3, *s*, H-27), 1.14 (**C**H_2, m, H-19α), 1.12 (**C**H_2, *m*, H-1α), 1.09 (**C**H_2, *m*, H-15α), 1.04 (**C**H_3, *s*, H-25), 0.99 (**C**H_3, *s*, H-24), 0.97 (**C**H_3, *s*, H-30), 0.93 (**C**H_3, *s*, H-23), 0.93 (**C**H_3, *s*, H-29), 0.89 (**C**H, *m*, H-5), 0.86 (**C**H_3, *s*, H-26) and **^{13}C-NMR spectrum** (MeOD, 125 MHz): 180.75 (C-28), 167.79 (C-9′), 159.87 (C-4′), 144.90 (C-13), 143.95 (C-7'), 129.75 (C-2′), 129.75 (C-6′), 125.77 (C-1′), 122.06 (C-12), 115.43 (C-3′), 115.43 (C-5′), 114.25 (C-8′), 80.90 (C-3), 55.41 (C-5), 48.45 (C-9), 46.32 (C-17), 45.90 (C-19), 41.51 (C-14), 41.38 (C-18), 39.16 (C-8), 37.95 (C-10), 37.58 (C-4), 37.40 (C-1), 33.53 (C-21), 32.51 (C-22), 32.45 (C-7), 32.18 (C-29), 30.23 (C-20), 27.46 (C-15), 27.26 (C-23), 25.00 (C-27), 23.29 (C-11), 23.12 (C-2), 22.68 (C-30), 22.59 (C-16), 17.97 (C-6), 16.34 (C-26), 15.94 (C-25), 14.53 (C-24)

^{1}H spectrum

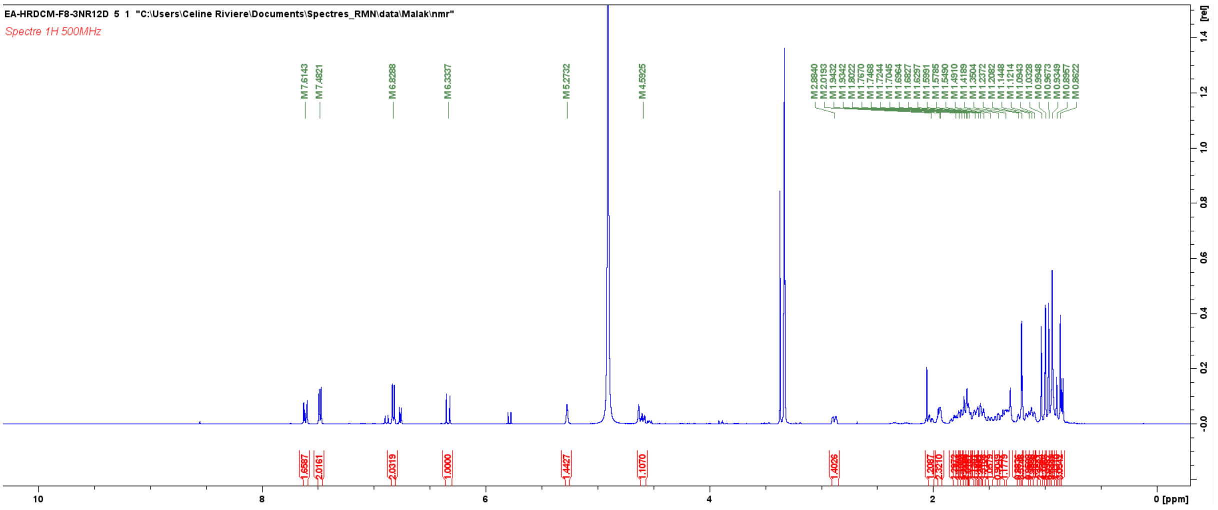

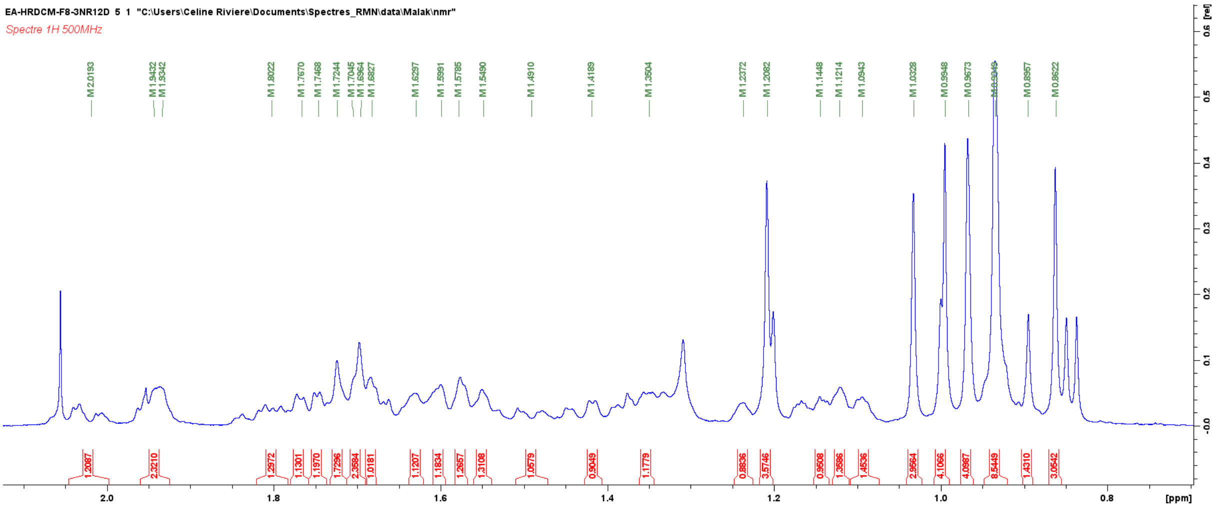

^{13}C spectrum

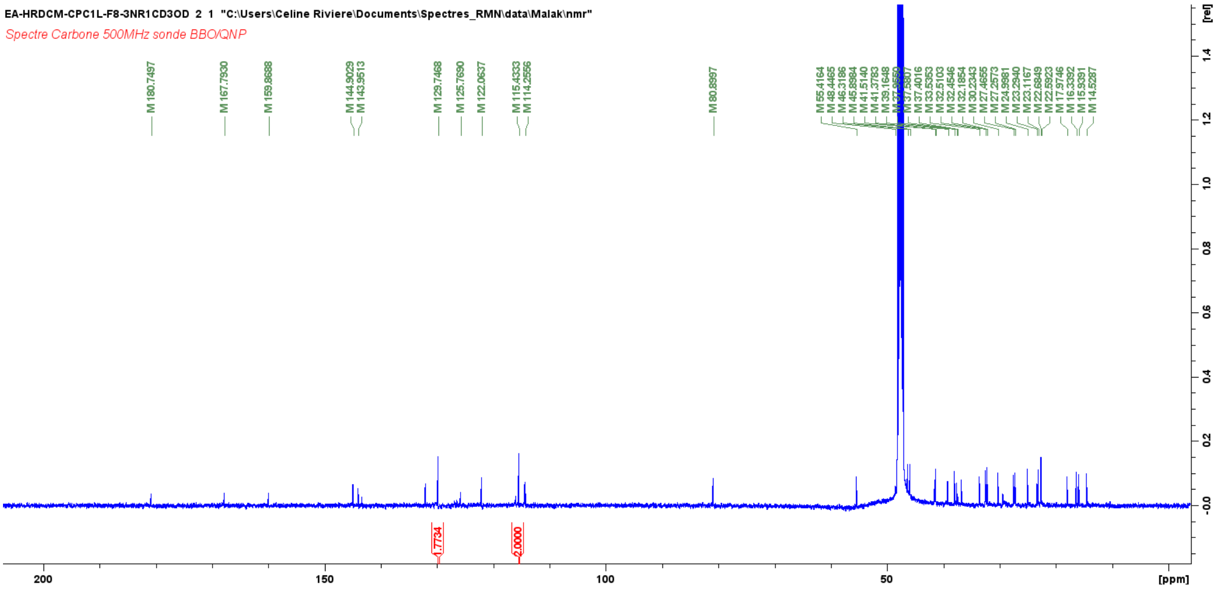

COSY spectrum

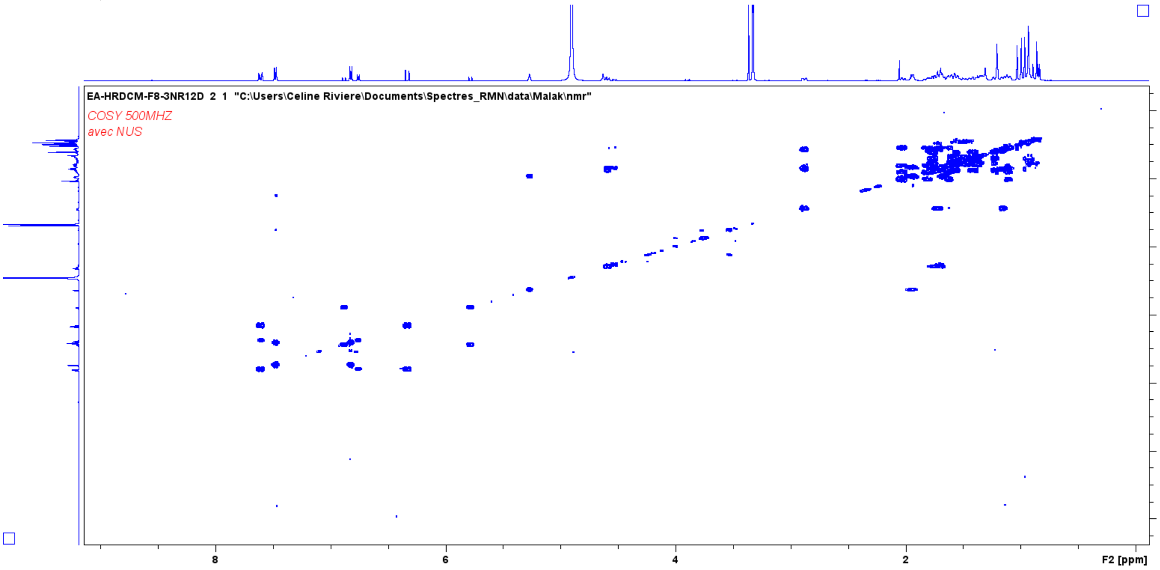

HSQC spectrum

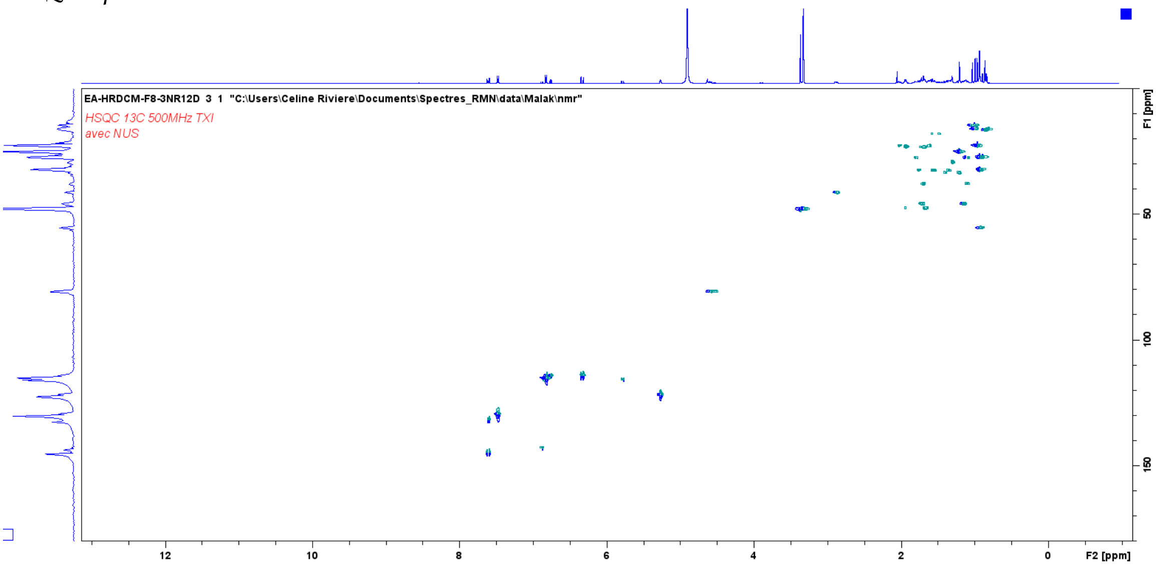

HMBC spectrum

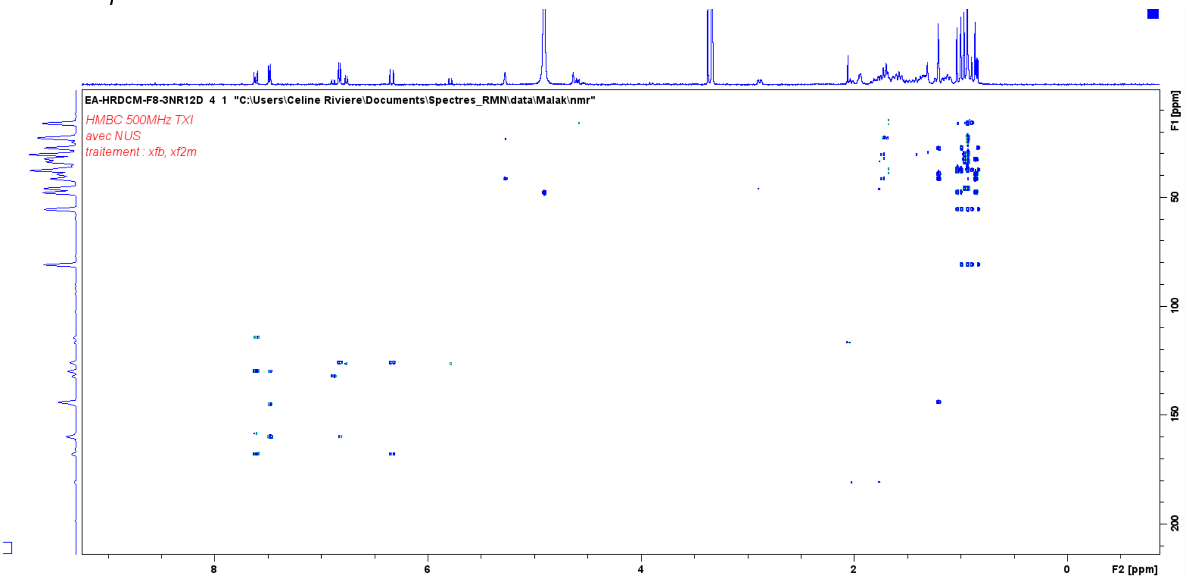

Supplement: Supplementary file 1 [file ijms-24-16617-s001.zip › ijms-2720107-supplementary.pdf]
